# Sixty Years After a Coal Mine Disaster: Serum Metabolomic Profiles in Older Adults with Long-Term Sequelae of Carbon Monoxide Poisoning: A Cross-Sectional Study

**DOI:** 10.3390/metabo16020126

**Published:** 2026-02-12

**Authors:** Eriko Baba, Hiroo Matsuse, Ryuki Hashida, Norika Matsukuma, Yuji Maki, Masayuki Omoto, Yoshio Takano, Makiko Motooka, Hiromichi Motooka

**Affiliations:** 1 Rehabilitation Center, Kurume University, 67 Asahi-Machi, Kurume 830-0011, Fukuoka, Japanhashida_ryuuki@kurume-u.ac.jp (R.H.); omoto_masayuki@med.kurume-u.ac.jp (M.O.); 2 Department of Physical Therapy, Osaka University of Arts Junior College, 4-8-70 Aramaki, Itami 664-0001, Hyogo, Japan; takano.yoshio@osaka-geidai.ac.jp; 3 Department of Neuropsychiatry, Kurume University Hospital, 67 Asahi-machi, Kurume 830-0011, Fukuoka, Japanhiromichi.motooka@hirocampus.com (H.M.)

**Keywords:** carbon monoxide poisoning, long-term sequelae, metabolomics, amino acids, ketone body, purine metabolism, BDNF, aging

## Abstract

Background: Survivors with chronic sequelae of carbon monoxide (CO) poisoning after the 1963 Miike–Mikawa coal mine disaster can exhibit persistent higher brain dysfunction in late life. We examined whether serum metabolic alterations remained detectable ~60 years later and assessed serum brain-derived neurotrophic factor (BDNF). Methods: In this cross-sectional case–control study, outpatients with chronic CO-poisoning sequelae (CO; n = 14) and former miners without CO exposure (CON; n = 16), all aged ≥ 75 years, underwent targeted serum metabolomics (1183 metabolites) and clinical assessments. Between-group differences were evaluated using Welch’s *t*-test, and age-matched propensity-score matching (1:1) served as a sensitivity analysis. BDNF was additionally compared using a linear regression/ analysis of covariancemodel adjusting for age and Mini–Mental State Examination (MMSE). Results: Relative to controls, the CO group showed higher valine, alanine, and betaine and lower 3-hydroxybutyric acid, inosine, and hypoxanthine; these contrasts persisted with concordant direction after matching. Serum BDNF was lower in the CO group (unadjusted trend) and was significantly reduced after age/MMSE adjustment (*p* = 0.0252). Exploratory correlations between clinical measures and selected metabolites/BDNF were attenuated after accounting for group. Conclusions: Six decades after exposure, chronic CO sequelae were associated with a reproducible serum profile combining amino-acid elevations with relative suppression of ketone-body and purine-related metabolites, suggesting enduring alterations in systemic substrate handling and bioenergetics. If replicated in larger cohorts, such signatures—potentially alongside BDNF—should be regarded as hypothesis-generating; biomarker development would require external validation, longitudinal tracking, and assessment of intervention responsiveness before any clinical use is considered.

## 1. Introduction

Carbon monoxide (CO) poisoning is a major cause of toxic hypoxic brain injury and can lead to persistent neuropsychiatric and cognitive sequelae long after the acute exposure [1]. The coal-dust explosion at the Miike–Mikawa coal mine on 9 November 1963 caused a mass CO exposure event in Japan, and long-term follow-up has highlighted the clinical and social importance of chronic higher brain dysfunction among surviving victims. During an afternoon shift change, runaway rail cars triggered coal-dust explosions deep underground; because the ventilation fan continued to operate, CO spread throughout the mine and trapped nearly 1400 workers far from the entrance. Rescue entry was delayed for ~2 h, resulting in 438 deaths from acute CO poisoning and 839 non-fatal poisoning cases, many requiring hospital admission [2]. Even in late life, affected individuals may continue to show reduced cognitive and functional status that cannot be fully attributed to aging alone [2,3]. Consistent with this prolonged functional impact, life-space mobility [4]—a patient-centered indicator of real-world movement range and frequency—was reported to be lower in older adults with Miike disaster-related CO-poisoning sequelae than in community-dwelling older adults and was associated with locomotor performance (gait speed and Timed Up and Go), suggesting that restricted daily activity is closely linked to reduced mobility capacity in this condition [5].

Mechanistically, chronic vulnerability after CO poisoning likely reflects a convergence of hypoxic injury, mitochondrial respiratory inhibition (classically via cytochrome c oxidase), and downstream oxidative and inflammatory cascades. Experimental and clinical evidence indicates that CO exposure can trigger redox stress and neuroinflammation, processes that may contribute to sustained bioenergetic dysregulation and chronic cognitive impairment [3,6]. Within this framework, brain-derived neurotrophic factor (BDNF) has emerged as a candidate mediator linking neuronal resilience with cellular energetics: BDNF– tropomyosin receptor kinase B (TrkB) signaling supports neuronal mitochondrial biogenesis and function and intersects with oxidative stress regulation and synaptic maintenance [7,8]. In line with these concepts, we previously reported lower circulating BDNF levels in patients with chronic CO-poisoning sequelae from the same source population [9].

From a systemic metabolic perspective, long-standing mitochondrial and inflammatory stress would be expected to influence substrate handling and energy-related pathways measurable in peripheral blood [10,11,12]. Altered amino-acid metabolism, including branched-chain amino acids, has been linked to impaired oxidative metabolism and broader metabolic dysregulation in other contexts [13]. Betaine, a key methyl donor in one-carbon metabolism, is responsive to cellular stress states that affect methylation capacity and redox balance [14]. In addition, ketone bodies [15,16,17] and purine turnover/salvage [18,19,20] metabolites are tightly coupled to mitochondrial adenosine triphosphate (ATP) dynamics and may serve as peripheral markers of altered bioenergetics. However, metabolomics evidence in long-term survivors with chronic CO-poisoning sequelae—particularly at very late time points approaching ~60 years after exposure—remains limited.

Therefore, this study aimed to characterize serum metabolomic differences between older adults with chronic CO-poisoning sequelae related to the Miike–Mikawa disaster and age-matched former miners without disaster-related CO exposure, and to identify a robust CO-associated metabolic signature. As hypothesis-generating secondary analyses, we explored associations between key metabolites and clinical measures of cognition, cognitive-related behavior, and life-space mobility, and evaluated serum BDNF as a biologically motivated marker at the intersection of neurotrophic support and metabolic regulation [7,9]. By integrating long-term clinical characterization with serum metabolomics, we sought to generate mechanistic hypotheses regarding persistent metabolic remodeling in chronic CO sequelae.

## 2. Materials and Methods

### 2.1. Study Design and Participants

In this cross-sectional study, we recruited former coal miners associated with the coal-dust explosion at the Miike–Mikawa coal mine (Omuta, Japan) on 9 November 1963. The CO group comprised outpatients with chronic sequelae of CO poisoning with higher brain dysfunction who were receiving follow-up care through a workers’ compensation-based aftercare program; eligible participants were identified from periodic post-disaster surveys and long-term clinical records. Acute-phase severity at the time of the 1963 disaster (including duration of impaired consciousness and whether acute care was inpatient or outpatient) was available from long-term records and is summarized in our prior cohort report [9] (Table 1 therein). The control (CON) group comprised former miners who worked in the same mine but were considered not to have been affected by the disaster; they were identified from the labor union roster and invited by telephone or postcard. The inclusion criterion was age ≥ 75 years; for the CO group, outpatient follow-up was additionally required. Exclusion criteria were neuropsychiatric disorders other than CO-related sequelae; a history of cerebrovascular disease with evident residual paralysis or cognitive impairment; severe comorbidities that would preclude study participation (e.g., end-stage heart failure, advanced cancer, or severe renal failure); and, in the control group, cases in which disaster-related CO exposure could not be ruled out. For the serum metabolomics analysis, we further excluded individuals receiving medications that could materially affect energy metabolism (antidiabetic agents, thyroid-related medications [thyroid hormone replacement or antithyroid drugs], or systemic oral corticosteroids).

**Figure 1:**
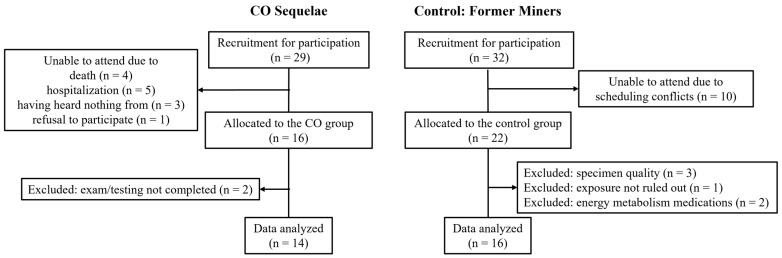
Participant flow diagram. In the serum metabolomics analysis, two participants in the CO group were excluded after allocation (no medical examination or incomplete neuropsychological testing), leaving 14 participants in the CO group for analysis. In the CON group, six participants were excluded (specimen quality issues, n = 3; possible disaster-related CO exposure, n = 1; use of medications that could affect energy metabolism, n = 2), resulting in 16 participants in the final CON analysis. CO, carbon monoxide; CO group, outpatients with chronic sequelae of CO poisoning and higher brain dysfunction; CON group, control group consisting of former miners without disaster-related CO exposure.

The serum metabolomics cohort was derived from the same participant pool as our prior Brain Disorders study [9]. After allocation, two participants in the CO group were excluded because they did not undergo the medical examination or could not complete the neuropsychological testing. Consequently, 14 participants in the CO group were included in the final analysis. In the CON group, six participants were excluded: three due to specimen quality issues (e.g., storage protocol deviations), one because disaster-related CO exposure could not be ruled out, and two due to use of medications that could materially affect energy metabolism. Ultimately, 14 participants in the CO group and 16 in the CON group were included in the serum metabolomics analysis (Figure 1). The study was approved by the Ethics Committee of Kurume University (No. 21107), conducted in accordance with the Declaration of Helsinki, and registered in UMIN (UMIN000053441). The dataset is publicly available in Harvard Dataverse (https://dataverse.harvard.edu/dataset.xhtml?persistentId=doi:10.7910/DVN/LMTK9R (accessed on 1 February 2026)). Written informed consent was obtained from all participants and their legally authorized representatives, as applicable.

### 2.2. Assessment

Assessments were performed as described previously [9] and are summarized here. Appendicular skeletal muscle mass was measured by a bioelectrical impedance analyzer (InBody S10, InBody Co., Ltd., Seoul, Republic of Korea) with participants in the supine position, and the skeletal muscle index (SMI) was calculated as appendicular skeletal muscle mass divided by height squared (kg/m^2^). Global cognitive function was evaluated using the Japanese version of the Mini–Mental State Examination (MMSE; total score 0–30) [21]. Attention and executive function were assessed using the standardized Japanese Trail Making Test (TMT-A and TMT-B) [22]; completion times (s) were recorded, and executive function was indexed as TMT-B minus TMT-A (TMT-B–A) [23].

### 2.3. Activities of Daily Living (ADLs)

ADLs were evaluated as described previously [9]. Independence was assessed using the Functional Independence Measure (FIM), yielding motor and cognitive subscores (motor: 13 items; cognitive: 5 items) [24]. Higher-brain-dysfunction-related ADLs were additionally assessed using the Cognitive-related Behavioral Assessment (CBA), which covers six domains (consciousness, emotion, attention, memory, judgment, and pathology); higher scores indicate better functioning [25].

### 2.4. Life-Space Mobility

Life-space mobility was assessed using the Life-Space Assessment (LSA) [4], which quantifies the extent and frequency of an individual’s mobility across progressively wider environments (from within the home to locations beyond one’s town) over the preceding 4 weeks. The LSA was administered as a structured interview by trained assessors, and a total score (0–120) was calculated according to the standard scoring algorithm, with higher scores indicating a wider life-space and greater community mobility.

### 2.5. BDNF

Blood sampling for BDNF was conducted as described previously [9]. Participants refrained from exercise for 48 h and fasted overnight (water permitted). Venous blood was collected from a forearm vein in the supine position between 09:00 and 10:00 to minimize diurnal effects, and sampling was performed on a separate day from functional testing. Serum was obtained by centrifugation, stored at −80 °C, and BDNF was quantified by an external laboratory (SRL, Inc., Tokyo, Japan) using an immunoassay (Human Free BDNF Quantikine ELISA Kit, R&D Systems, Inc., Minneapolis, MN, USA).

### 2.6. Serum Metabolomics

Serum metabolomics was outsourced to Human Metabolome Technologies, Inc. (Tsuruoka, Yamagata, Japan; HMT). Frozen serum aliquots were shipped to HMT on dry ice and maintained under frozen conditions until analysis. Sample preparation and CE–MS measurements were performed by HMT using a standardized capillary electrophoresis–mass spectrometry workflow [26]. Briefly, serum was deproteinized with methanol containing internal standards, followed by liquid–liquid partitioning with water and chloroform, centrifugation, and filtration through a 5 kDa cut-off filter to remove proteins. The filtrate was vacuum-concentrated and reconstituted in water containing reference compounds prior to capillary electrophoresis–time-of-flight mass spectrometry (CE–TOFMS) analysis. Raw mass spectrometrydata were processed by extracting metabolite-derived peaks and managing each peak using a unique peak identifier (ID). Peak IDs include a mode-specific prefix (e.g., C for cation mode and A for anion mode in CE-based measurements). Putative metabolite identities were annotated by matching measured mass-to-charge ratios (*m*/*z*) and migration times (MTs) against HMT’s in-house library/annotation table generated from authentic standards. Quantitative outputs are reported as Relative Area (relative concentration), defined as peak areas corrected for analytical sensitivity and sample amount using internal standards and predefined coefficients. Peaks not detected are reported as N.D. (not detected) in the dataset. The targeted panel comprised 1183 annotated metabolites (Supplementary Table S6). In the quantitative output returned by HMT, metabolites are reported when a corresponding peak was detected in at least one sample; metabolites with no detected peak across all samples are not reported. Accordingly, 246 annotated metabolites with at least one detected Relative Area value were available for downstream analyses; the list of tested metabolites with detection statistics and between-group comparisons is provided in Supplementary Table S5. Because CE–MS-based panels preferentially capture polar/ionic metabolites, pathway coverage is uneven (e.g., relatively limited lipid coverage); therefore, null findings for lipid-related analytes should be interpreted within the analytical scope of this platform.

### 2.7. Statistical Analysis

This study targeted a rare cohort of long-term survivors with chronic sequelae of CO poisoning 60 years after the Miike–Mikawa coal mine disaster and was conducted as an exploratory, hypothesis-generating analysis given the limited prior metabolomics evidence in this setting. For statistical testing, we evaluated all annotated metabolites reported by HMT (i.e., metabolites for which a corresponding peak was detected in at least one sample). N.D. and N.A. were treated as missing values. For univariate analyses, between-group differences (CO vs. CON) were evaluated for each metabolite using Welch’s *t*-test. To aid interpretation in the context of multiple testing, Benjamini–Hochberg false discovery rate (BH-FDR) *q* values were additionally calculated across the tested metabolites and are reported in Supplementary Table S5. As a sensitivity/robustness analysis, age-matched propensity-score matching (1:1) was performed, and the same univariate comparisons (Welch’s *t*-tests) were repeated in the matched cohort. Robustness was primarily assessed by concordant effect direction (CO > CON or CO < CON) between the unmatched and matched analyses; in addition, standardized effect sizes (Hedges’ g) were calculated for each comparison and considered when interpreting the magnitude and consistency of between-group differences across cohorts. For key metabolites, results from the unmatched and matched cohorts are presented side-by-side as the between-group mean difference (CO − CON; or ratio), 95% confidence interval, *p* value, and Hedges’ g (defined such that positive values indicate higher concentrations in the CO group). For baseline characteristics, cognitive/functional measures, and serum BDNF (Table 1), between-group differences were assessed using Welch’s *t*-test, and Hedges’ g was additionally calculated to quantify standardized effect sizes. As a supplementary analysis to aid comparability with our prior study from the same source population, we assessed between-group differences in serum BDNF using an analysis of covariance (ANCOVA)/linear regression model adjusting for age and MMSE (Supplementary Table S1); this analysis was included to contextualize BDNF within the metabolomics-focused objectives of the present work. Group differences in clinical variables (TMT, MMSE, FIM, LSA, CBA, SMI, and body mass index (BMI)) were also assessed using Welch’s *t*-test. Correlation analyses were restricted to metabolites that remained significant after age-matched propensity-score matching (1:1) and were evaluated using Spearman’s rank correlation coefficients. Group-adjusted partial correlations (controlling for CO/CON group) were additionally examined as a supplementary analysis (Supplementary Table S2). To characterize inter-metabolite relationships within the key metabolic signature, we additionally assessed unadjusted Spearman correlations among the six key metabolites that remained significant after matching (valine, alanine, betaine, 3-hydroxybutyric acid, inosine, and hypoxanthine). As a robustness check, we also evaluated group-adjusted residual-based correlations among these metabolites (i.e., partial Spearman correlations controlling for CO vs. CON group) to determine whether inter-metabolite associations persisted after accounting for between-group differences (Supplementary Table S3). Data are presented as mean ± standard deviation (SD) unless otherwise specified. Given the exploratory metabolomics setting and multiple testing, we report nominal two-sided *p* values alongside effect sizes, and provide BH-FDR *q* values (Supplementary Table S5) to aid interpretation.

## 3. Results

Participant characteristics are summarized in Table 1. The CO and control groups were broadly comparable in age, BMI, and SMI (all *p* > 0.14), with small-to-moderate standardized differences (Hedges’ *g*) for these baseline variables (e.g., age *g* = 0.12; BMI *g* = −0.57; SMI *g* = −0.39). Relative to controls, the CO group showed poorer cognitive and functional performance, including lower MMSE and FIM-cognitive scores and reduced LSA, along with lower CBA scores and longer TMT-B completion times (all *p* < 0.05). These impairments were accompanied by large effect sizes across key outcomes (e.g., MMSE *g* = −1.13; FIM-cognitive *g* = −1.07; LSA *g* = −1.13; CBA *g* = −1.32; TMT-B *g* = 0.87). Serum BDNF tended to be lower in the CO group, although the difference did not reach statistical significance (*p* = 0.0729); nonetheless, the standardized effect size suggested a moderate between-group difference (Hedges’ *g* = −0.66). In a supplementary ANCOVA/linear regression model adjusting for age and MMSE, the between-group difference in serum BDNF was statistically significant (*p* = 0.02524; Supplementary Table S1).

Of the 1183 annotated metabolites in the targeted panel, 246 were reported by HMT because a corresponding peak was detected in at least one sample; metabolites with no detected peak in any sample were not reported. These 246 metabolites were available for downstream analyses (see Supplementary Table S5; the analyte list is provided in Supplementary Table S6). Between-group differences in serum metabolites are presented in Table 2 (effect sizes [Hedges’ g] and 95% CIs are shown). In the unmatched cohort, the metabolites shown in Table 2A exhibited nominal between-group differences (Welch’s *t*-test *p* < 0.05), and BH-FDR *q* values across all tested metabolites are provided in Supplementary Table S5. These contrasts showed concordant directions in the age-matched propensity-score-matched cohort (Table 2B), supporting the robustness of the key metabolic pattern in sensitivity analysis. No metabolite survived BH-FDR correction (*q* < 0.05) across metabolites with available *p* values (Supplementary Table S5); therefore, these findings should be interpreted as exploratory and hypothesis-generating.

We next explored clinical–metabolite associations, focusing on metabolites that showed nominal between-group differences after matching (Table 3). *p* values are nominal and provided for descriptive purposes; because correlations can be driven by between-group separation, group-adjusted partial correlations are reported in Supplementary Table S2. In unadjusted Spearman analyses, LSA was inversely correlated with alanine (ρ = −0.4219, *p* = 0.0202), and FIM-cognitive was likewise inversely correlated with alanine (ρ = −0.5124, *p* = 0.0038). In contrast, FIM-cognitive was positively correlated with 3-hydroxybutyric acid, inosine, and hypoxanthine (ρ = 0.4977, *p* = 0.0051; ρ = 0.4763, *p* = 0.0186; and ρ = 0.5016, *p* = 0.0047, respectively). Serum BDNF also showed an inverse association with alanine (ρ = −0.3838, *p* = 0.0363). MMSE was inversely correlated with betaine (ρ = −0.3619, *p* = 0.0494) and positively correlated with hypoxanthine (ρ = 0.3641, *p* = 0.0479). CBA was inversely correlated with alanine (ρ = −0.3614, *p* = 0.0497) but positively correlated with 3-hydroxybutyric acid (ρ = 0.5069, *p* = 0.0043). Valine showed no significant correlations, although its association with FIM-cognitive showed a trend (*p* = 0.0563). TMT-B was not significantly correlated with any metabolite (all *p* ≥ 0.18) (see Supplementary Table S4 for the full unadjusted Spearman’s correlation results between serum metabolites and clinical measures, including BDNF).

To assess whether these unadjusted correlations reflected within-group relationships beyond differences between CO and control participants, we performed supplementary partial correlation analyses adjusting for group (CO vs. CON). After group adjustment, the observed associations were generally attenuated and no longer statistically significant (Supplementary Table S2), suggesting that the unadjusted clinical–metabolite correlations were influenced, at least in part, by between-group differences related to chronic CO sequelae.

Finally, to better describe the internal structure of the metabolic signature, we examined inter-metabolite correlations among the six key metabolites (Table 4). In unadjusted analyses, valine was strongly positively correlated with alanine (ρ = 0.6721, *p* < 0.0001), and inosine was strongly positively correlated with hypoxanthine (ρ = 0.6730, *p* = 0.0003). Valine was inversely correlated with inosine (ρ = −0.4435, *p* = 0.0300), and alanine was inversely correlated with 3-hydroxybutyric acid (ρ = −0.4082, *p* = 0.0251), whereas other pairwise correlations were not statistically significant.

As a robustness check, we further evaluated group-adjusted residual-based correlations controlling for CO vs. CON (Supplementary Table S3). The positive correlations between valine and alanine (ρ = 0.5991, *p* = 0.0005) and between inosine and hypoxanthine (ρ = 0.5177, *p* = 0.0096) remained statistically significant, and alanine also showed a positive residual correlation with hypoxanthine (ρ = 0.4672, *p* = 0.0092). In contrast, the inverse associations of valine with inosine and alanine with 3-hydroxybutyric acid were attenuated and were no longer statistically significant after adjustment.

## 4. Discussion

### 4.1. Principal Findings and Clinical Context

In this exploratory metabolomics study of long-term survivors with chronic CO-poisoning sequelae ~60 years after the Miike–Mikawa coal mine disaster, we identified a reproducible serum signature (valine, alanine, betaine, 3-hydroxybutyric acid, inosine, and hypoxanthine) that showed concordant direction across unmatched and propensity-score-matched analyses. Serum BDNF was also lower in the CO group and remained significant after age/MMSE adjustment, consistent with our prior report from the same source population [9].

### 4.2. Amino Acids and One-Carbon Metabolism: Long-Term Substrate Handling Shift

From a metabolic perspective, the CO group showed higher concentrations of multiple amino acids (e.g., valine and alanine) and higher betaine, suggesting a long-standing shift in systemic substrate handling rather than a purely age-related change. This interpretation is consistent with evidence that mitochondrial/inflammatory stress is reflected in peripheral blood metabolite profiles across populations and disease states [10,12]. Elevated alanine may reflect sustained transamination and gluconeogenic substrate cycling [27], suggesting reliance on amino-acid-derived carbon and nitrogen flux under chronic metabolic stress. Increased valine (a branched-chain amino acid) may reflect altered amino-acid catabolism linked to impaired oxidative metabolism and broader metabolic dysregulation in other settings [13]. The concomitant increase in betaine is notable because betaine is a key methyl donor in one-carbon metabolism and is responsive to cellular stress states that influence methylation capacity and redox balance [14]. While dietary intake can influence circulating amino acids and betaine [28,29], the consistent direction of between-group differences after matching and the parallel neurofunctional impairments in the CO group support the interpretation that these findings may reflect chronic disease-related metabolic remodeling; however, nutritional factors (and physical activity) cannot be excluded.

### 4.3. Ketone Bodies and Purine-Related Metabolites: Bioenergetic Flexibility

In contrast to the amino-acid elevations, the CO group showed lower 3-hydroxybutyric acid and lower purine/energy-related metabolites (including inosine and hypoxanthine, and in the full targeted panel also adenosine diphosphate), a pattern that is compatible with reduced metabolic flexibility and disturbed bioenergetics. Because CO can directly impair mitochondrial respiration—classically via inhibition of cytochrome c oxidase—downstream alterations in oxidative phosphorylation, redox stress, and inflammatory signaling may persist or re-emerge over time, potentially contributing to chronic neurocognitive vulnerability [30,31,32]. Although the targeted panel included multiple acylcarnitines and glycerophosphocholine, these lipid-related metabolites did not differ between groups in the present study (Supplementary Table S5). Within the analytical scope of the present panel, these null findings do not exclude mitochondrial dysfunction but suggest that the long-term CO-associated signature observed here is more strongly reflected in amino-acid, ketone-body, and purine/energy-related pathways than in persistent accumulation of acylcarnitines or glycerophosphocholine [11,33]. Lower 3-hydroxybutyric acid may indicate reduced ketone availability and/or utilization—an alternative energy substrate with recognized signaling roles that intersect with oxidative stress pathways [15]. Meanwhile, inosine and hypoxanthine lie within purine turnover/salvage pathways that are tightly coupled to ATP dynamics; shifts in these metabolites can therefore be interpreted as markers of altered energy metabolism and nucleotide handling under chronic stress [18]. In this context, evidence from primary respiratory-chain disease supports that systemic bioenergetic impairment can yield coordinated changes in classic and non-classic cardiometabolic analytes detectable in peripheral biofluids, reinforcing the plausibility that the observed ketone/purine suppression reflects downstream consequences of long-standing mitochondrial perturbation [12].

### 4.4. BDNF in Chronic CO Sequelae: A Neurotrophic–Bioenergetic Marker

In the present metabolomics cohort, serum BDNF was lower in the CO sequelae group, although the unadjusted comparison did not reach statistical significance (*p* = 0.0729); nevertheless, the standardized effect size indicated a moderate between-group difference (Hedges’ g = −0.66). Importantly, when we applied an age- and MMSE-adjusted ANCOVA/linear regression model—aligned with the analytic strategy used in our prior report—the group difference became statistically significant (*p* = 0.02524; Supplementary Table S1), supporting the presence of a CO-associated reduction in circulating BDNF after accounting for key covariates. Consistent with this finding, in our prior study drawn from the same source population, BDNF was more clearly reduced in the CO sequelae group, with a comparable moderate unadjusted standardized effect size (Hedges’ g ≈ −0.65) [9]. The initial non-significance in the unadjusted comparison of the current analysis is plausibly explained by limited statistical power after restricting to the metabolomics subset (including additional exclusions for medications that could affect energy metabolism), together with the inherent biological variability of circulating BDNF and reliance on a single time-point measurement. Mechanistically, BDNF–TrkB signaling has been shown to support neuronal mitochondrial biogenesis and function (e.g., via peroxisome proliferator-activated receptor gamma coactivator 1-alpha -related pathways and mitochondrial respiration), providing a biologically plausible link between neurotrophic support, oxidative stress, and cellular energetics [7,8]. Given that CO poisoning can trigger hypoxic–oxidative and inflammatory cascades that may perturb mitochondrial homeostasis [3,6], the directionally consistent and covariate-supported reduction in BDNF in this metabolomics cohort remains biologically coherent and provides a rationale for examining how BDNF relates to the CO-associated metabolite signature. Moreover, population data linking peripheral blood mitochondrial indices to both circulating metabolites and inflammatory markers provide additional support for a systemic axis connecting mitochondrial stress, inflammation, and peripheral metabolic readouts [10].

### 4.5. Exploratory Phenotype–Metabolite Associations and Robustness Checks

In exploratory, hypothesis-generating Spearman correlation analyses in this small-sample cohort, functional, mobility, and cognitive behavioral measures showed several associations with the CO-related metabolite pattern and serum BDNF. In unadjusted analyses, LSA and cognitive functional status (FIM-cognitive) were inversely related to alanine, whereas FIM-cognitive showed positive associations with 3-hydroxybutyric acid and purine-related metabolites (inosine and hypoxanthine). Serum BDNF also exhibited an inverse association with alanine. In addition, the CBA, which may reflect the severity of higher brain dysfunction, showed associations with selected metabolites (e.g., an inverse association with alanine and a positive association with 3-hydroxybutyric acid). These findings are presented as preliminary signals to guide future hypothesis testing in chronic CO sequelae, rather than implying causal relationships.

When we accounted for group membership (CO vs. CON) using supplementary group-adjusted partial/residual-based correlation approaches, the unadjusted clinical–metabolite associations were attenuated and were no longer statistically significant. This pattern suggests that at least part of the apparent correlations in the pooled cohort may have been driven by between-group separation—i.e., the presence of chronic CO sequelae—rather than reflecting stable within-group (individual-level) relationships. Importantly, this does not negate potential biological links, but emphasizes that clinical–metabolite coupling should be interpreted cautiously in a two-group design with limited sample size. In this context, our use of sensitivity analyses (propensity-score matching and group-adjusted correlation checks) provides a transparent robustness framework and helps delineate which associations are more likely to reflect group-level differences versus within-group covariation.

Beyond clinical correlations, the inter-metabolite relationship structure among the six robust metabolites provides additional mechanistic clues. The strong positive correlation between valine and alanine supports a coherent amino-acid module that may reflect shared upstream regulation (e.g., altered substrate utilization and amino-acid handling under chronic metabolic stress). Likewise, the positive correlation between inosine and hypoxanthine is consistent with coordinated purine turnover/salvage dynamics, aligning with the notion that energy-related nucleotide handling is perturbed in chronic CO sequelae. Notably, several of these inter-metabolite correlations persisted in group-adjusted residual analyses (e.g., valine–alanine and inosine–hypoxanthine), suggesting covariation that is not solely attributable to between-group differences and thus may represent a more intrinsic metabolic linkage within the observed signature. Conversely, pairwise correlations that diminished after group adjustment may be more dependent on group separation and should be interpreted more conservatively.

### 4.6. Implications and Future Directions

From a clinical standpoint, the present findings suggest that serum metabolite profiles—potentially in combination with BDNF—may capture a disease-relevant facet of chronic CO-related higher brain dysfunction even decades after exposure. For reader accessibility, a pathway-based schematic integrating the amino-acid/one-carbon, ketone-body, purine/energy-related findings and BDNF are provided in Supplementary Figure S1. Importantly, these observations should be regarded as hypothesis-generating and not as established biomarkers. If validated, such profiles could be explored as complementary research signals alongside conventional assessments (e.g., FIM-cognitive and LSA) as hypothesis-generating signatures reflecting systemic bioenergetic and neurotrophic dysregulation. Such validation would require (i) replication in an external cohort, (ii) longitudinal sampling to assess stability and temporal association with clinical trajectories, and (iii) intervention studies (e.g., exercise/nutrition) to test responsiveness and clinical relevance. Moreover, because amino-acid handling, ketone availability, and purine-related metabolism are potentially modifiable, these pathways may provide testable targets for future intervention studies (e.g., exercise or nutritional strategies), where metabolite/BDNF changes could serve as exploratory readouts. However, given the exploratory design, small sample size, and cross-sectional nature, these clinical applications should be viewed as provisional and require replication and longitudinal/mechanistic confirmation.

### 4.7. Limitations

This study has several limitations. First, the sample size was small and derived from a rare long-term survivor cohort, which limits statistical power and generalizability and increases the risk of false-positive findings. Second, the cross-sectional design precludes causal inference and does not allow evaluation of temporal dynamics of metabolite and BDNF changes. Third, although we used age-matched propensity-score matching and additional adjusted analyses, residual confounding (e.g., comorbidities, diet, physical activity, and unmeasured medication effects) may remain, and multiple-comparison issues are a concern even within a targeted panel. In particular, we did not collect quantitative dietary intake (e.g., recent protein intake, supplementation) or objective physical-activity measures. Because circulating amino acids, betaine, and ketone bodies are sensitive to nutrition and activity, residual confounding by these factors remains possible. Finally, metabolomic measures were obtained from peripheral blood and therefore may not directly reflect brain-specific metabolism or mitochondrial function; mechanistic interpretation should be considered provisional until replicated and complemented by longitudinal and experimental studies.

## 5. Conclusions

In this exploratory study of long-term survivors with chronic CO-poisoning sequelae ~60 years after the Miike–Mikawa coal mine disaster, we identified a reproducible serum metabolite pattern—higher amino-acid-related metabolites with lower ketone-body and purine-related metabolites—and covariate-supported lower circulating BDNF. Although causal inference is not possible, these findings provide hypothesis-generating evidence of persistent systemic metabolic remodeling in late-life CO sequelae and motivate larger, longitudinal and mechanistic studies to clarify clinical relevance.

## Figures and Tables

**Table 1:** Baseline characteristics, cognitive/functional measures, and serum BDNF levels in the CO and control groups.

Variable	CO Group	Control Group	Mean Difference (95% CI)	Hedges’ g	*p*-Value
Age (years)	83.5 ± 2.8	83.2 ± 2.3	−0.3 (−2.3, 1.6)	0.115	0.7432
BMI (kg/m^2^)	22.7 ± 2.5	24.4 ± 3.2	1.7 (−0.6, 3.9)	−0.571	0.1415
SMI (kg/m^2^)	6.8 ± 0.7	7.1 ± 0.8	0.3 (−0.3, 0.9)	−0.386	0.2840
BDNF (pg/mL)	18,373.6 ± 8130.7	23,945.6 ± 8206.0	5572 (−554, 11,698)	−0.663	0.0729
MMSE score (points)	21.2 ± 6.9	27.3 ± 3.2	6.1 (1.9, 10.3)	−1.130	0.0070
TMT-A (s)	99.7 ± 51.1	72.7 ± 30.7	−27.0 (−62.1, 8.1)	0.634	0.1232
TMT-B (s)	222.7 ± 95.6	143.9 ± 81.5	−78.7 (−150.0, −7.5)	0.868	0.0318
Motor-FIM score	86.7 ± 7.6	90.6 ± 0.5	3.8 (−0.5, 8.2)	−0.731	0.0799
Cognitive-FIM score	30.1 ± 6.4	34.9 ± 0.3	4.9 (1.2, 8.6)	−1.070	0.0138
LSA (points)	64.7 ± 21.5	87.6 ± 18.1	22.9 (7.9, 37.9)	−1.128	0.0043
CBA score	25.0 ± 3.7	28.6 ± 1.1	3.6 (1.5, 5.8)	−1.323	0.0029

Values are mean ± SD. Mean difference is defined as CO − CON, with 95% confidence intervals (CIs) reported for the mean difference. Hedges’ g (standardized mean difference; small-sample corrected) was calculated using the pooled SD; positive values indicate higher values in the CO group. *p* values were obtained using Welch’s *t*-test for between-group comparisons. Abbreviations: CO, carbon monoxide; CON, control; CO group, outpatients with chronic sequelae of CO poisoning and higher brain dysfunction; CON group, control group consisting of former miners without disaster-related CO exposure; BMI, body mass index; SMI, skeletal muscle mass index; BDNF, brain-derived neurotrophic factor; MMSE, Mini–Mental State Examination; TMT, Trail Making Test; FIM, Functional Independence Measure; CBA, Cognitive-related Behavioral Assessment; LSA, Life-Space Assessment.

**Table 2:** (**A**) Between-group differences in serum amino acids and related metabolites in the CO and control groups in the unmatched cohort. (**B**) Between-group differences in serum amino acids and related metabolites in the CO and control groups in the age-matched propensity-score-matched cohort (1:1).

**(A)**						
**Metabolites**	**CO Group** **(Unmatched, n = 14)**	**Control Group** **(Unmatched, n = 16)**	**Mean Difference (95% CI)** **(Unmatched)**	**Hedges’ g** **(Unmatched)**	***p*-Value** **(Unmatched)**	
Valine (μM)	256.0 ± 50.0	221.0 ± 27.0	34.9 (3.8, 66.0)	0.865	0.0298	
Alanine (μM)	423.0 ± 101.0	316.0 ± 51.0	107.3 (44.7, 169.8)	1.330	0.002	
Arginine (μM)	80.0 ± 46.0	77.0 ± 19.0	22.3 (4.4, 40.2)	0.085	0.0167	
Glycine (μM)	234.0 ± 61.0	186.0 ± 29.0	48.5 (11.2, 85.8)	1.001	0.0136	
Lysine (μM)	276.0 ± 75.0	223.0 ± 31.0	52.4 (7.0, 97.9)	0.922	0.0263	
Sarcosine (μM)	3.5 ± 1.0	2.6 ± 0.8	0.9 (0.3, 1.6)	0.975	0.0092	
Betaine (μM)	67 ± 11.0	58.0 ± 13.0	9.5 (0.4, 18.7)	0.723	0.0415	
3-Hydroxybutyric acid (μM)	43.0 ± 18.0	100.0 ± 60.0	−57.5 (−90.5, −24.5)	−1.216	0.0018	
ADP (μM)	5.3 ± 1.8	7.2 ± 2.4	−1.8 (−3.5, −0.1)	−0.863	0.0345	
Inosine (μM)	1.1 ± 0.3	1.8 ± 1.1	−0.7 (−1.3, −0.1)	−0.820	0.0329	
Hypoxanthine (μM)	7.0 ± 2.7	14.0 ± 7.0	−7.3 (−11.2, −3.4)	−1.251	0.0009	
**(B)**						
**Metabolites**	**CO Group** **(Matched, n = 9)**	**Control Group (Matched, n = 9)**	**Mean Difference (95% CI)** **(Matched)**	**Hedges’ g** **(Matched)**	***p*-Value** **(Matched)**	**Direction Concordant**
Valine (μM)	256.8 ± 46.5	217.2 ± 18.2	39.7 (2.7, 76.6)	1.068	0.0377	Yes
Alanine (μM)	420.0 ± 91.2	315 ± 48.0	104.1 (29.3, 178.9)	1.372	0.0104	Yes
Arginine (μM)	102.4 ± 30.9	76.8 ± 19.5	25.6 (−0.6, 51.9)	0.944	0.0544	Yes
Glycine (μM)	214.3 ± 47.2	189.1 ± 34.7	25.2 (−16.5, 66.9)	0.579	0.2162	Yes
Lysine (μM)	272.2 ± 84.2	228.6 ± 22.6	43.6 (−22.0, 109.1)	0.674	0.1675	Yes
Sarcosine (μM)	3.2 ± 1.1	2.6 ± 0.7	0.6 (−0.3, 1.5)	0.620	0.2010	Yes
Betaine (μM)	68.1 ± 9.9	55.8 ± 12.1	12.3 (1.2, 23.4)	1.060	0.0323	Yes
3-Hydroxybutyric acid (μM)	41.9 ± 14.3	107.3 ± 70.2	−65.4 (−119.7, −11.1)	−1.230	0.0237	Yes
ADP (μM)	5.7 ± 1.9	7.4 ± 3.2	−1.7 (−4.6, 1.2)	−0.615	0.2195	Yes
Inosine (μM)	0.999 ± 0.1	2.044 ± 1.4	−1.0 (−2.1, −0.0)	−1.003	0.0493	Yes
Hypoxanthine (μM)	7.1 ± 2.4	15.7 ± 8.5	−8.6 (−15.3, −1.9)	−1.311	0.0171	Yes

Values are mean ± SD (μM). Unmatched comparisons were performed in the full cohort (CO, n = 14; CON, n = 16) using Welch’s *t*-test. Mean difference is defined as CO − CON with 95% confidence intervals (CIs). Hedges’ g (standardized mean difference; small-sample corrected) was calculated using the pooled SD; positive values indicate higher concentrations in the CO group. *p* values are nominal (two-sided Welch’s *t*-test). BH-FDR *q* values across all tested metabolites are provided in Supplementary Table S5; findings should be interpreted as exploratory. Mean difference is defined as CO − CON with 95% confidence intervals (CIs). Hedges’ g (standardized mean difference; small-sample corrected) was calculated using the pooled SD; positive values indicate higher concentrations in the CO group. ‘Direction concordant’ indicates whether the sign of the mean difference was consistent with that observed in the unmatched cohort. *p* values are nominal (two-sided Welch’s *t*-test). BH-FDR *q* values across all tested metabolites are provided in Supplementary Table S5; emphasis is placed on concordant directions and effect sizes in this sensitivity analysis. Abbreviations: CO, carbon monoxide; CO group, outpatients with chronic sequelae of CO poisoning and higher brain dysfunction; Control group, control group consisting of former miners without disaster-related CO exposure; SD, standard deviation; CI, confidence interval; BH-FDR, Benjamini–Hochberg false discovery rate; ADP, adenosine diphosphate.

**Table 3:** Unadjusted Spearman’s rank correlations between key amino acids/metabolites and clinical measures (including serum BDNF).

	BDNF	TMT-B	Cognitive-FIM	MMSE	LSA	CBA
Valine	0.2494 (0.1838)	0.1613 (0.4121)	−0.3521 (0.0563)	−0.2675 (0.1530)	−0.1157 (0.5426)	−0.2279 (0.2257)
Alanine	−0.3838 (0.0363)	0.2386 (0.2215)	−0.5124 (0.0038)	−0.3299 (0.0750)	−0.4219 (0.0202)	−0.3614 (0.0497)
Betaine	−0.1146 (0.5466)	0.2547 (0.1908)	−0.1507 (0.4267)	−0.3619 (0.0494)	−0.1902 (0.3141)	−0.1971 (0.2964)
3-Hydroxybutyric acid	0.1061 (0.5768)	−0.2605 (0.1807)	0.4977 (0.0051)	0.2766 (0.1389)	0.2491 (0.1844)	0.5069 (0.0043)
Inosine	0.2183 (0.3056)	−0.1864 (0.3944)	0.4763 (0.0186)	0.1447 (0.5000)	0.0889 (0.6794)	0.2563 (0.2266)
Hypoxanthine	0.3362 (0.0693)	−0.1490 (0.4492)	0.5016 (0.0047)	0.3641 (0.0479)	0.2676 (0.1529)	0.2676 (0.0723)

Cells show Spearman’s rank correlation coefficient, with the corresponding two-sided *p* value in parentheses [ρ (*p*)]. Metabolites are expressed in μM and serum BDNF in pg/mL. Missing values were handled by pairwise deletion; therefore, the sample size may vary across pairs. *p* values are nominal and provided for descriptive purposes. Because correlations can be driven by between-group separation, group-adjusted partial correlations are reported in Supplementary Table S2. Abbreviations: FIM-cognitive, cognitive subscale of the Functional Independence Measure; LSA, Life-Space Assessment; MMSE, Mini-Mental State Examination; TMT-B, Trail Making Test Part B; CBA, Cognitive Battery Assessment; BDNF, brain-derived neurotrophic factor.

**Table 4:** Unadjusted Spearman’s rank correlation matrix among key serum metabolites (valine, alanine, betaine, 3-hydroxybutyric acid, inosine, and hypoxanthine).

	Valine	Alanine	Betaine	3-Hydroxybutyric Acid	Inosine	Hypoxanthine
Valine	-	0.6721 (<0.0001)	−0.0398 (0.8345)	−0.1043 (0.5832)	−0.4435 (0.0300)	−0.0959 (0.6142)
Alanine	0.6721 (<0.0001)	-	0.2303 (0.2209)	−0.4082 (0.0251)	−0.2496 (0.2396)	−0.0785 (0.6800)
Betaine	−0.0398 (0.8345)	0.2303 (0.2209)	-	−0.2432 (0.1954)	−0.0078 (0.9710)	−0.2854 (0.1263)
3-Hydroxybutyric acid	−0.1043 (0.5832)	−0.4082 (0.0251)	−0.2432 (0.1954)	-	0.0870 (0.6862)	0.2752 (0.1411)
Inosine	−0.4435 (0.0300)	−0.2496 (0.2396)	−0.0078 (0.9710)	0.0870 (0.6862)	-	0.6730 (0.0003)
Hypoxanthine	−0.0959 (0.6142)	−0.0785 (0.6800)	−0.2854 (0.1263)	0.2752 (0.1411)	0.6730 (0.0003)	-

Cells show Spearman’s rank correlation coefficient, with the corresponding two-sided *p* value in parentheses [ρ (*p*)]. Metabolites are expressed in μM. Correlations were calculated in the full cohort (CO and CON combined); missing values were handled by pairwise deletion, and thus sample sizes may vary across pairs. *p* values are nominal and provided for descriptive purposes. Because correlations can be driven by between-group separation in this two-group design, these unadjusted correlations should be interpreted cautiously; group-adjusted partial correlations are reported in Supplementary Table S2.

## Data Availability

The dataset is publicly available in Harvard Dataverse (https://dataverse.harvard.edu/dataset.xhtml?persistentId=doi:10.7910/DVN/LMTK9R (accessed on 1 February 2026)).

## Supplementary Materials

The following supporting information can be downloaded at: https://www.mdpi.com/article/10.3390/metabo16020126/s1, Table S1: ANCOVA/linear regression for serum BDNF adjusted for age and MMSE; Table S2: Group-adjusted partial Spearman’s correlations between selected metabolites and clinical measures; Table S3: Group-adjusted (residual-based) Spearman’s rank correlations among key serum metabolites (valine, alanine, betaine, 3-hydroxybutyric acid, inosine, and hypoxanthine); Table S4: Unadjusted Spearman’s rank correlations between serum metabolites and clinical measures (including BDNF); Table S5: Annotated metabolites reported by HMT (detected in at least one sample): detection rates, group-wise summary statistics, and between-group comparisons; Table S6: Targeted metabolite panel (n = 1183): annotated metabolite list; Figure S1: Integrated pathway schematic of metabolomic findings and BDNF.

## References

[1] Sönmez B.M., İşcanlı M.D., Parlak S., Doğan Y., Ulubay H.G., Temel E. (2018). Delayed neurologic sequelae of carbon monoxide intoxication. Turk. J. Emerg. Med..

[2] Mimura K., Harada M., Sumiyoshi S., Toya G., Takagi M., Fujita E., Takata A., Tatetsu S. (2023). Long-term effects of carbon monoxide poisoning at Miike coal mine: A 33-year follow-up study. Undersea Hyperb. Med..

[3] Weaver L.K. (2009). Clinical practice. Carbon monoxide poisoning. N. Engl. J. Med..

[4] Baker P.S., Bodner E.V., Allman R.M. (2003). Measuring life-space mobility in community-dwelling older adults. J. Am. Geriatr. Soc..

[5] Matsuse H., Hashida R., Iwanaga S., Baba E., Takano Y., Shiba N. (2022). Life-Space Mobility in Elderly Patients with Sequelae of Carbon Monoxide Poisoning 55 Years Later from Miike Explosion of Coal Dust. J. Kurume Med. Assoc..

[6] Arya A.K., Sethuraman K., Waddell J., Cha Y.S., Liang Y., Bhopale V.M., Thom S.R. (2025). Carbon monoxide poisoning triggers neurodegeneration and neuroinflammation in an aging model of cognitive impairment. Sci. Adv..

[7] Cheng A., Wan R., Yang J.-L., Kamimura N., Son T.G., Ouyang X., Luo Y., Okun E., Mattson M.P. (2012). Involvement of PGC-1α in the formation and maintenance of neuronal dendritic spines. Nat. Commun..

[8] Swain M., Soman S.K., Tapia K., Dagda R.Y., Dagda R.K. (2023). Brain-derived neurotrophic factor protects neurons by stimulating mitochondrial function through protein kinase A. J. Neurochem..

[9] Baba E., Hashida R., Takano Y., Maki Y., Tajima H., Motooka M., Motooka H., Matsuse H. (2025). Blood brain-derived neurotrophic factor levels in patients with sequelae of carbon-monoxide poisoning 60 years after a coal-dust explosion: A cross-sectional study. Brain Disord..

[10] Knez J., Marrachelli V.G., Cauwenberghs N., Winckelmans E., Zhang Z., Thijs L., Brguljan-Hitij J., Plusquin M., Delles C., Monleon D. (2017). Peripheral blood mitochondrial DNA content in relation to circulating metabolites and inflammatory markers: A population study. PLoS ONE.

[11] Johansson P.I., Nakahira K., Rogers A.J., McGeachie M.J., Baron R.M., Fredenburgh L.E., Harrington J., Choi A.M.K., Christopher K.B. (2018). Plasma mitochondrial DNA and metabolomic signatures of acute respiratory distress syndrome. Crit. Care.

[12] Thompson Legault J., Strittmatter L., Tardif J., Sharma R., Tremblay-Vaillancourt V., Aubut C., Boucher G., Clish C.B., Cyr D., Daneault C. (2015). A metabolic signature of mitochondrial dysfunction revealed through a monogenic form of Leigh syndrome. Cell Rep..

[13] Newgard C.B., An J., Bain J.R., Muehlbauer M.J., Stevens R.D., Lien L.F., Haqq A.M., Shah S.H., Arlotto M., Slentz C.A. (2009). A branched-chain amino acid-related metabolic signature that differentiates obese and lean humans and contributes to insulin resistance. Cell Metab..

[14] Ueland P.M., Holm P.I., Hustad S. (2005). Betaine: A key modulator of one-carbon metabolism and homocysteine status. Clin. Chem. Lab. Med..

[15] Newman J.C., Verdin E. (2017). β-Hydroxybutyrate: A Signaling Metabolite. Annu. Rev. Nutr..

[16] Cotter D.G., Schugar R.C., Crawford P.A. (2013). Ketone body metabolism and cardiovascular disease. Am. J. Physiol. Heart Circ. Physiol..

[17] Puchalska P., Crawford P.A. (2021). Metabolic and Signaling Roles of Ketone Bodies in Health and Disease. Annu. Rev. Nutr..

[18] Furuhashi M. (2020). New insights into purine metabolism in metabolic diseases: Role of xanthine oxidoreductase activity. Am. J. Physiol. Endocrinol. Metab..

[19] Wu Z., Bezwada D., Cai F., Harris R.C., Ko B., Sondhi V., Pan C., Vu H.S., Nguyen P.T., Faubert B. (2024). Electron transport chain inhibition increases cellular dependence on purine transport and salvage. Cell Metab..

[20] Farthing D.E., Farthing C.A., Xi L. (2015). Inosine and hypoxanthine as novel biomarkers for cardiac ischemia: From bench to point-of-care. Exp. Biol. Med..

[21] Ideno Y., Takayama M., Hayashi K., Takagi H., Sugai Y. (2012). Evaluation of a Japanese version of the Mini-Mental State Examination in elderly persons. Geriatr. Gerontol. Int..

[22] Llinàs-Reglà J., Vilalta-Franch J., López-Pousa S., Calvó-Perxas L., Torrents Rodas D., Garre-Olmo J. (2017). The Trail Making Test. Assessment.

[23] Sánchez-Cubillo I., Periáñez J.A., Adrover-Roig D., Rodríguez-Sánchez J.M., Ríos-Lago M., Tirapu J., Barceló F. (2009). Construct validity of the Trail Making Test: Role of task-switching, working memory, inhibition/interference control, and visuomotor abilities. J. Int. Neuropsychol. Soc..

[24] Keith R.A., Granger C.V., Hamilton B.B., Sherwin F.S. (1987). The functional independence measure: A new tool for rehabilitation. Adv. Clin. Rehabil..

[25] Maki Y., Morita A., Makizako H. (2023). Association between the Cognitive-Related Behavioral Assessment Severity Stage and Activities of Daily Living Required for Discharge to Home in Patients with Stroke: A Cross-Sectional Study. Int. J. Environ. Res. Public Health.

[26] Soga T. (2007). Capillary electrophoresis-mass spectrometry for metabolomics. Methods Mol. Biol..

[27] Felig P. (1973). The glucose-alanine cycle. Metabolism.

[28] Cavallaro N.L., Garry J., Shi X., Gerszten R.E., Anderson E.J., Walford G.A. (2016). A pilot, short-term dietary manipulation of branched chain amino acids has modest influence on fasting levels of branched chain amino acids. Food Nutr. Res..

[29] Atkinson W., Slow S., Elmslie J., Lever M., Chambers S.T., George P.M. (2009). Dietary and supplementary betaine: Effects on betaine and homocysteine concentrations in males. Nutr. Metab. Cardiovasc. Dis..

[30] Alonso J.R., Cardellach F., López S., Casademont J., Miró O. (2003). Carbon monoxide specifically inhibits cytochrome c oxidase of human mitochondrial respiratory chain. Pharmacol. Toxicol..

[31] Garrabou G., Inoriza J.M., Morén C., Oliu G., Miró Ò., Martí M.J., Cardellach F. (2011). Mitochondrial injury in human acute carbon monoxide poisoning: The effect of oxygen treatment. J. Environ. Sci. Health Part C.

[32] Angelova P.R., Myers I., Abramov A.Y. (2023). Carbon monoxide neurotoxicity is triggered by oxidative stress induced by ROS production from three distinct cellular sources. Redox Biol..

[33] McCoin C.S., Knotts T.A., Adams S.H. (2015). Acylcarnitines—old actors auditioning for new roles in metabolic physiology. Nat. Rev. Endocrinol..

