# Supplementary material for: Sixty Years After a Coal Mine Disaster: Serum Metabolomic Profiles in Older Adults with Long-Term Sequelae of Carbon Monoxide Poisoning: A Cross-Sectional Study
Source: Metabolites. 2026 Feb 12;16(2):126. doi: 10.3390/metabo16020126 (PMC12943369; doi:10.3390/metabo16020126)
Supplement: Supplementary file 1 [file metabolites-16-00126-s001.zip › metabolites-4124646-supplementary/Revised Supplementary files/revised S2.pdf]

Supplementary Table S2. Group-adjusted partial Spearman’s correlations between selected metabolites and clinical measure

	BDNF	Cognitive–FIM	LSA	MMSE	TMT-B	CBA
**Valine (µM)**	-0.1389(0.4642)	0.0531(0.7804)	0.0721(0.7048)	-0.0482(0.8003)	0.0396(0.8145)	-0.0197(0.9178)
**Alanine (µM)**	-0.2512(0.1806)	-0.0529(0.7813)	-0.2318(0.2177)	0.0316(0.8685)	0.0053(0.9786)	-0.0690(0.7170)
**Betaine (µM)**	0.0099(0.9587)	0.0099(0.9587)	-0.0492(0.7961)	-0.5199(0.2982)	0.1072(0.5873)	0.0382(0.8413)
**3-Hydroxybutyric acid (µM)**	-0.1266(0.5049)	-0.1130(0.5521)	-0.0309(0.8714)	-0.1518(0.4232)	-0.0147(0.9410)	0.2575(0.1695)
**Inosine (µM)**	0.0331(0.8780)	0.2834(0.1797)	-0.2146(0.3139)	-0.1627(0.4475)	0.1275(0.5621)	0.0397(0.8540)
**Hypoxanthine (µM)**	0.1550(0.4133)	0.0896(0.6377)	-0.0582(0.7601)	-0.1074(0.5723)	0.2414(0.2159)	-0.0202(0.9155)

Cells show group-adjusted partial Spearman’s rank correlation coefficient, ρ, with the corresponding two-sided p value in parentheses [ρ (p)], controlling for group (CO vs. CON). Correlation analyses were restricted to metabolites that showed nominal between-group differences with concordant direction in the propensity-score–matched analysis (1:1). Partial Spearman’s correlations were computed by correlating residuals of rank-transformed variables after regression on group. Metabolites are expressed in µM and serum BDNF in pg/mL. Missing values were handled by pairwise deletion; therefore, the sample size may vary across pairs. P values are nominal and provided for descriptive purposes. Because correlations can be influenced by between-group separation, results should be interpreted cautiously in this small-sample two-group design. Abbreviations: FIM-cognitive, cognitive subscale of the Functional Independence Measure; LSA, Life-Space Assessment; MMSE, Mini-Mental State Examination; TMT-B, Trail Making
