# Supplementary material for: Sixty Years After a Coal Mine Disaster: Serum Metabolomic Profiles in Older Adults with Long-Term Sequelae of Carbon Monoxide Poisoning: A Cross-Sectional Study
Source: Metabolites. 2026 Feb 12;16(2):126. doi: 10.3390/metabo16020126 (PMC12943369; doi:10.3390/metabo16020126)
Supplement: Supplementary file 1 [file metabolites-16-00126-s001.zip › metabolites-4124646-supplementary/Revised Supplementary files/revised S3.pdf]

Supplementary Table S3. Group-adjusted (residual-based) Spearman's rank correlations among key serum metabolites (valine, alanine, betaine, 3-hydroxybutyric acid, inosine, and hypoxanthine).

	Valine	Alanine	Betaine	3-Hydroxybutyric acid	Inosine	Hypoxanthine
**Valine**	-	0.5991 (0.0005)	-0.2053 (0.2766)	0.1619 (0.3928)	-0.2046 (0.3375)	0.2306 (0.2202)
**Alanine**	0.5991 (0.0005)	-	0.0403 (0.8326)	-0.1226 (0.5187)	0.2299 (0.2798)	0.4672 (0.0092)
**Betaine**	-0.2053 (0.2766)	0.0403 (0.8326)	-	-0.0367 (0.8473)	0.2092 (0.3265)	-0.0367 (0.8473)
**3-Hydroxybutyric acid**	0.1619 (0.3928)	-0.1226 (0.5187)	-0.0367 (0.8473)	-	-0.2529 (0.2331)	-0.2081 (0.2698)
**Inosine**	-0.2046 (0.3375)	0.2299 (0.2798)	0.2092 (0.3265)	-0.2529 (0.2331)	-	0.5177 (0.0096)
**Hypoxanthine**	0.2306 (0.2202)	0.4672 (0.0092)	-0.0367 (0.8473)	-0.2081 (0.2698)	0.5177 (0.0096)	-

Cells show group-adjusted partial Spearman's rank correlation coefficient, ρ, with the corresponding two-sided p value in parentheses [ρ (p)], controlling for group (CO vs. CON). Partial Spearman's correlations were computed by correlating residuals of rank-transformed variables after regression on group. Metabolites are expressed in μM. Missing values were handled by pairwise deletion; therefore, the sample size may vary across pairs. P values are nominal and provided for descriptive purposes; no multiple-testing
