# Supplementary material for: Sixty Years After a Coal Mine Disaster: Serum Metabolomic Profiles in Older Adults with Long-Term Sequelae of Carbon Monoxide Poisoning: A Cross-Sectional Study
Source: Metabolites. 2026 Feb 12;16(2):126. doi: 10.3390/metabo16020126 (PMC12943369; doi:10.3390/metabo16020126)
Supplement: Supplementary file 1 [file metabolites-16-00126-s001.zip › metabolites-4124646-supplementary/Revised Supplementary files/revised S4.pdf]

Supplementary Table S4. Unadjusted Spearman's rank correlations between serum metabolites and clinical measures (including BDNF)

	Age	BMI	SMI	Motor–FIM	Cognitive–FIM	CBA	TMT-A	TMT-B	TMT-B-A	MMSE	LSA	BDNF
**Alanine (µM)**	0.2395(0.2024)	-0.2047(0.278)	0.1347(0.5028)	-0.2858(0.1257)	-0.5124(0.00038)	-0.3614(0.00497)	0.0849(0.6676)	0.2386(0.2215)	0.2162(0.2692)	-0.3299(0.075)	-0.4219(0.0202)	-0.3838(0.0363)
**Arginine (µM)**	-0.0433(0.8201)	-0.3423(0.0641)	-0.2367(0.2345)	-0.2033(0.2813)	-0.4373(0.0517)	-0.3403(0.0657)	0.1684(0.3917)	0.1706(0.3853)	0.0898(0.6496)	-0.3956(0.00305)	-0.3133(0.0919)	-0.2182(0.2466)
**Betaine (µM)**	-0.0953(0.6165)	-0.1545(0.4148)	-0.0009(0.9964)	-0.199(0.2917)	-0.1507(0.4267)	-0.1971(0.2964)	0.3641(0.0568)	0.2547(0.1908)	0.1363(0.4892)	-0.3619(0.0494)	-0.1902(0.3141)	-0.1146(0.5466)
**Glycine (µM)**	0.1309(0.4904)	-0.3216(0.0831	-0.1008(0.6171)	-0.1494(0.4306)	-0.2361(0.209)	-0.3981(0.0293)	0.0871(0.6596)	-0.0364(0.854)	-0.1007(0.6101)	-0.2406(0.2002)	-0.2522(0.1788)	-0.2952(0.1132)
**Valine (µM)**	-0.1827(0.0888)	-0.114(0.5486)	0.1133(0.5736)	0.0223(0.9069)	-0.3521(0.0563)	-0.2279(0.2257)	0.0348(0.8606)	0.1613(0.4121)	0.156(0.428)	-0.2675(0.153)	-0.1157(0.5426)	-0.2494(0.1838)
**Sarcosine (µM)**	-0.1858(0.3256)	-0.216(0.2516)	-0.0107(0.9577)	-0.2206(0.2414)	-0.3679(0.0455)	-0.419(0.0212)	0.0298(0.8802)	0.0323(0.8703)	0.0038(0.9846)	-0.2838(0.1286)	-0.3576(0.0523)	-0.2289(0.2237)
**Lysine (µM)**	0.0251(0.8954)	-0.2158(0.2521)	0.0625(0.7569)	-0.3068(0.7433)	-0.3674(0.0458)	-0.1668(0.3784)	-0.0172(0.9306)	0.1123(0.5694)	0.0794(0.6881)	-0.1595(0.4)	-0.1654(0.3828)	-0.3068(0.0992)
**3-Hydroxybutyric acid (µM)**	0.0738(0.6982)	0.1174(0.5368)	0.0156(0.9384)	0.3568(0.053)	0.4977(0.0051)	0.5069(0.0043)	-0.0988(0.6168)	-0.2605(0.1807)	-0.2605(0.1305)	0.2766(0.1389)	0.2491(0.1844)	0.1061(0.5768)
**ADP (uM)**	-0.104(0.101)	0.0523(0.7957)	0.2261(0.2261)	0.0499(0.8046)	0.2859(0.1483)	0.4327(0.0242)	0.00725(0.7248)	-0.1184(0.5446)	-0.094(0.6478)	0.0513(0.7993)	0.0992(0.6225(	0.5537(0.0027)
**Hypoxanthine (µM)**	0.0296(0.8767)	0.067(0.7249)	0.1528(0.4467)	0.2576(0.1693)	0.5016(0.0047)	0.3329(0.0723)	-0.0805(0.6839)	-0.149(0.4492)	-0.1067(0.5888)	0.3641(0.0479)	0.2676(0.1529)	0.3362(0.0693)
**Inosine (µM)**	-0.2905(0.1684)	0.0192(0.9292)	0.1344(0.5551)	0.2862(0.1752)	0.4763(0.0186)	0.2563(0.2266)	0.0544(0.8053)	-0.1864(0.3944)	-0.1864(0.391)	0.1447(0.5)	0.0889(0.6794)	0.2183(0.3056)

Cells show Spearman's ρ, with the corresponding two-sided p value in parentheses [ρ (p)]. P values are nominal and provided for descriptive purposes. Because correlations can be driven by between-group separation in this two-group comparison, group-adjusted partial correlations are reported in Supplementary Table S2. Metabolites are expressed in µM and serum BDNF in pg/mL. Missing values were handled by pairwise deletion; therefore, the sample size may vary across pairs. Abbreviations: FIM-cognitive, cognitive subscale of the Functional Independence Measure; LSA, Life-Space Assessment; MMSE, Mini-Mental State Examination; TMT, Trail Making Test; TMT-B–A, difference between TMT Part B and Part A; CBA, Cognitive Battery Assessment; BMI, body mass index; SMI, skeletal muscle mass index; BDNF, brain-derived neurotrophic factor.
