# Supplementary material for: Sixty Years After a Coal Mine Disaster: Serum Metabolomic Profiles in Older Adults with Long-Term Sequelae of Carbon Monoxide Poisoning: A Cross-Sectional Study
Source: Metabolites. 2026 Feb 12;16(2):126. doi: 10.3390/metabo16020126 (PMC12943369; doi:10.3390/metabo16020126)
Supplement: Supplementary file 1 [file metabolites-16-00126-s001.zip › metabolites-4124646-supplementary/Revised Supplementary files/revised S5.pdf]

Supplementary Table S5. Annotated metabolites reported by HMT (detected in at least one sample): detection rates, group-wise summary statistics, and between-group comparisons

Peak ID	Metabolite name	PubChem CID	HMDB ID	m/z	MT/RT	n_CO	CO detection rate	CO mean	CO SD	CO mean±SD	n_CON	CON detection rate	CON mean	CON SD	CON mean±SD	n_total	Overall detection rate	Mean ratio (CO/CON)	Hedges' g	Welch p	BH-FDR q
C_0063	1-Methyl-4-imidazoleacetic acid	75810	HMDB0002820	141.0655	6.461744	12	85.7%	1.3263E-04	6.4225E-05	0.0001326 ± 6.422e-05	16	100.0%	1.2098E-04	4.3384E-05	0.000121 ± 4.338e-05	28	93.3%	1.0963E+00	0.213	0.5941	0.8442
C_0164	1-Methyladenosine	27476	HMDB0003331	282.119	7.723433	11	78.6%	6.2199E-05	1.1487E-05	6.22e-05 ± 1.149e-05	13	81.3%	6.2618E-05	1.7569E-05	6.262e-05 ± 1.757e-05	24	80.0%	9.9331E-01	-0.027	0.9448	0.9812
C_0096	1-Methylhistidine<br>3-Methylhistidine	9210564969	HMDB0000001<br>HMDB0000479	170.0924	5.906221	14	100.0%	2.9860E-03	1.6256E-03	0.002986 ± 0.00162[illegible]	16	100.0%	2.2648E-03	8.8811E-04	0.002265 ± 0.0008881	30	100.0%	1.3184E+00	0.546	0.1553	0.5642
C_0057	1-Methylnicotinamide	457	HMDB0000699	137.071	5.801609	9	64.3%	1.2316E-04	1.6012E-04	0.0001232 ± 0.0001601	13	81.3%	9.8116E-05	3.5668E-05	9.812e-05 ± 3.567e-05	22	73.3%	1.2552E+00	0.230	0.6561	0.8561
C_0064	1H-Imidazole-4-propionic acid	10105257		141.0658	6.359287	1	7.1%	7.7298E-05		7.73e-05	0	0.0%			N.A.	1	3.3%				
A_0091	2,3-Diphosphoglyceric acid	186004	HMDB0001294	264.9522	13.04071	0	0.0%			N.A.	1	6.3%	3.1840E-03		0.003184	1	3.3%				
A_0039	2,6-Dihydroxybenzoic acid	9338	HMDB0013676	153.0206	8.740426	2	14.3%	1.1244E-04	8.0007E-05	0.0001124 ± 8.001e-05	0	0.0%			N.A.	2	6.7%				
C_0015	2-Aminoisobutyric acid<br>2-Aminobutyric acid	61196657	HMDB0001906<br>HMDB0000452	104.0705	7.491085	14	100.0%	8.0222E-03	2.4750E-03	0.008022 ± 0.002475	16	100.0%	8.4494E-03	2.3218E-03	0.008449 ± 0.002322	30	100.0%	9.4944E-01	-0.174	0.6313	0.8543
C_0085	2-Aminooctanoic acid	69522	HMDB0000991	160.1329	8.349263	5	35.7%	8.6045E-05	3.2576E-05	8.605e-05 ± 3.258e-05	3	18.8%	8.2531E-05	4.5264E-06	8.253e-05 ± 4.526e-06	8	26.7%	1.0426E+00	0.114	0.8233	0.9274
A_0037	2-Deoxyribonic acid	11528367	HMDB0000366	149.0455	7.067344	12	85.7%	3.6794E-04	1.0058E-04	0.0003679 ± 0.0001006	11	68.8%	3.7019E-04	1.1746E-04	0.0003702 ± 0.0001175	23	76.7%	9.9392E-01	-0.020	0.9613	0.9812
A_0017	2-Ethylhydracrylic acid<br>5-Hydroxypentanoic acid	18897925945	HMDB0000396<br>HMDB0061927	117.0555	7.341432	13	92.9%	3.1380E-04	1.1282E-04	0.0003138 ± 0.0001128	15	93.8%	3.2032E-04	1.1017E-04	0.0003203 ± 0.0001102	28	93.3%	9.7965E-01	-0.057	0.8787	0.9622
A_0026	2-Hydroxy-4-methylvaleric acid<br>2-Hydroxy-3-methylvaleric acid	439960164623	HMDB0000624<br>HMDB0000317	131.0709	7.284566	8	57.1%	2.2726E-04	1.5551E-04	0.0002273 ± 0.0001555	8	50.0%	1.7231E-04	3.8004E-05	0.0001723 ± 3.8e-05	16	53.3%	1.3189E+00	0.459	0.3606	0.7265
A_0008	2-Hydroxybutyric acid	440864	HMDB0000008	103.0399	7.939372	14	100.0%	3.5597E-03	1.4510E-03	0.00356 ± 0.001451	16	100.0%	4.0930E-03	1.3152E-03	0.004093 ± 0.001315	30	100.0%	8.6970E-01	-0.376	0.3037	0.7091
A_0009	2-Hydroxyisobutyric acid	11671	HMDB0000729	103.0399	8.01927	9	64.3%	4.0952E-04	1.5916E-04	0.0004095 ± 0.0001592	9	56.3%	3.5936E-04	1.3146E-04	0.0003594 ± 0.0001315	18	60.0%	1.1396E+00	0.327	0.4770	0.7715
C_0074	2-Methylthiazolidine-4-carboxylic acid	160736	HMDB0246657	148.0427	10.87296	13	92.9%	5.8484E-04	3.0259E-04	0.0005848 ± 0.0003026	16	100.0%	4.1058E-04	2.7666E-04	0.0004106 ± 0.0002767	29	96.7%	1.4244E+00	0.587	0.1218	0.4973
A_0034	2-Oxoglutaric acid	51	HMDB0000208	145.0139	14.33608	4	28.6%	6.9687E-04	4.4427E-04	0.0006969 ± 0.0004443	3	18.8%	7.9369E-04	5.7873E-04	0.0007937 ± 0.0005787	7	23.3%	8.7801E-01	-0.162	0.8222	0.9274
A_0013	2-Oxoisovaleric acid	49	HMDB0000019	115.0397	8.216558	14	100.0%	8.3143E-04	2.8307E-04	0.0008314 ± 0.0002831	16	100.0%	9.7950E-04	3.7656E-04	0.0009795 ± 0.0003766	30	100.0%	8.4883E-01	-0.428	0.2306	0.6612
A_0060	2-Phosphoglyceric acid	439278	HMDB0003391	184.9852	13.30451	0	0.0%			N.A.	1	6.3%	3.8774E-04		0.0003877	1	3.3%				
A_0047	3-(4-Hydroxyphenyl)propionic acid<br>2-(4-Hydroxyphenyl)propionic acid<br>3-(3-Hydroxyphenyl)propionic [illegible]	1039410252691124	[illegible]<br>HMDB0041683<br>HMDB0000375<br>HMDB0062590	165.0551	6.910551	2	14.3%	1.9109E-04	4.1030E-05	0.0001911 ± 4.103e-05	0	0.0%			N.A.	2	6.7%				
C_0025	3-Amino-2-piperidone	5200225	HMDB0000323	115.0864	5.977212	7	50.0%	9.5742E-05	4.3686E-05	9.574e-05 ± 4.369e-05	6	37.5%	9.4640E-05	4.5314E-05	9.464e-05 ± 4.531e-05	13	43.3%	1.0117E+00	0.023	0.9654	0.9812
C_0017	3-Aminobutyric acid	10932		104.0707	6.238717	10	71.4%	2.3787E-04	4.5717E-05	0.0002379 ± 4.572e-05	10	62.5%	1.6401E-04	1.4834E-05	0.000164 ± 1.483e-05	20	66.7%	1.4504E+00	2.082	0.0005	0.0615
C_0014	3-Aminoisobutyric acid	64956	HMDB0003911	104.0705	6.158221	14	100.0%	9.1659E-04	8.0600E-04	0.0009166 ± 0.000806	16	100.0%	1.2329E-03	1.0177E-03	0.001233 ± 0.001018	30	100.0%	7.4347E-01	-0.333	0.3509	0.7247
A_0007	3-Hydroxybutyric acid	441	HMDB0000011,HMDB0000357,HMDB0000442	103.0399	7.763687	14	100.0%	2.8293E-03	1.2081E-03	0.002829 ± 0.001208	16	100.0%	6.6326E-03	3.9536E-03	0.006633 ± 0.003954	30	100.0%	4.2657E-01	-1.216	0.0018	0.0648
C_0044	3-Hydroxyproline	150779	HMDB0002113	132.0654	8.407645	12	85.7%	9.6646E-05	2.8269E-05	9.665e-05 ± 2.827e-05	13	81.3%	9.5342E-05	2.7248E-05	9.534e-05 ± 2.725e-05	25	83.3%	1.0137E+00	0.045	0.9076	0.9688
A_0079	3-Indoxylsulfuric acid	10258	HMDB0000682	212.0022	7.748545	14	100.0%	1.1649E-03	4.5291E-04	0.001165 ± 0.0004529	16	100.0%	1.4941E-03	9.2263E-04	0.001494 ± 0.0009226	30	100.0%	7.7965E-01	-0.431	0.2193	0.6511
A_0061	3-Phosphoglyceric acid	439183	HMDB0000807	184.9859	13.57495	4	28.6%	3.4992E-04	9.2472E-05	0.0003499 ± 9.247e-05	4	25.0%	7.6266E-04	1.1400E-03	0.0007627 ± 0.00114	8	26.7%	4.5882E-01	-0.444	0.5220	0.8025
C_0068	4-Guanidinobutyric acid	500	HMDB0003464	146.0923	6.497152	5	35.7%	8.8963E-05	4.8380E-05	8.896e-05 ± 4.838e-05	5	31.3%	1.1682E-04	6.1520E-05	0.0001168 ± 6.152e-05	10	33.3%	7.6154E-01	-0.455	0.4503	0.7480
A_0023	4-Methyl-2-oxovaleric acid<br>3-Methyl-2-oxovaleric acid<br>2-Oxohexanoic acid	7047159664	HMDB0000695<br>HMDB0000491<br>HMDB0001864	129.0554	7.775907	14	100.0%	7.2717E-03	2.9374E-03	0.007272 ± 0.002937	16	100.0%	7.5697E-03	1.7600E-03	0.00757 ± 0.00176	30	100.0%	9.6063E-01	-0.122	0.7438	0.8875
C_0040	4-Oxopyrrolidine-2-carboxylic acid	107541		130.0499	8.494119	14	100.0%	3.9172E-04	1.5714E-04	0.0003917 ± 0.0001571	16	100.0%	3.5066E-04	1.7375E-04	0.0003507 ± 0.0001738	30	100.0%	1.1171E+00	0.240	0.5024	0.7877
A_0012	4-Oxovaleric acid	11579	HMDB0000720	115.0394	7.877341	4	28.6%	2.2972E-04	1.1836E-04	0.0002297 ± 0.0001184	4	25.0%	1.3186E-04	4.4381E-05	0.0001319 ± 4.438e-05	8	26.7%	1.7421E+00	0.952	0.1996	0.6257
C_0039	5-Amino-3,4-dihydro-2H-pyrrole-2-carboxylic acid	13894665		129.0658	6.839743	14	100.0%	4.4074E-03	1.8025E-03	0.004407 ± 0.001802	16	100.0%	4.6520E-03	2.5309E-03	0.004652 ± 0.002531	30	100.0%	9.4742E-01	-0.107	0.7608	0.8875
C_0090	5-Hydroxylysine	3032849	HMDB0000450	163.1077	5.635004	10	71.4%	8.1098E-05	4.7983E-05	8.11e-05 ± 4.798e-05	12	75.0%	5.3078E-05	1.3778E-05	5.308e-05 ± 1.378e-05	22	73.3%	1.5279E+00	0.798	0.1036	0.4942
A_0021	5-Methylfuran-2-carboxylic acid	74710	HMDB0059735	125.0249	7.739345	1	7.1%	1.5915E-04		0.0001592	0	0.0%			N.A.	1	3.3%				
A_0022	5-Oxoproline	7405	HMDB0000267	128.0351	7.708599	14	100.0%	3.1923E-03	1.5791E-03	0.003192 ± 0.001579	16	100.0%	2.8692E-03	4.7146E-04	0.002869 ± 0.0004715	30	100.0%	1.1126E+00	0.278	0.4723	0.7715
C_0093	7-Methylguanine	11361	HMDB0000897	166.0726	6.460359	8	57.1%	4.9991E-05	1.7752E-05	4.999e-05 ± 1.775e-05	12	75.0%	5.6207E-05	1.1457E-05	5.621e-05 ± 1.146e-05	20	66.7%	8.8942E-01	-0.418	0.3999	0.7265
C_0095	7-Methylxanthine	68374	HMDB0001991	167.0573	15.42663	4	28.6%	1.3075E-04	3.1118E-05	0.0001308 ± 3.112e-05	0	0.0%			N.A.	4	13.3%				

A_0041	8-Hydroxyoctanoic acid-1 3-Hydroxyoctanoic acid-1	6982026613	HMDB0010722	159.1025	6.614174	3	21.4%	8.4339E-04	2.4176E-04	0.0008434 ± 0.0002418	0	0.0%			N.A.	3	10.0%				
A_0042	8-Hydroxyoctanoic acid-2 3-Hydroxyoctanoic acid-2	6982026613	HMDB0010722	159.1031	6.714098	3	21.4%	6.5516E-04	2.6674E-04	0.0006552 ± 0.0002667	2	12.5%	1.3561E-04	2.3297E-05	0.0001356 ± 2.33e-05	5	16.7%	4.8310E+00	1.732	0.0761	0.4441
C_0055	Adenine	190	HMDB0000034	136.0614	5.998394	0	0.0%			N.A.	2	12.5%	4.6659E-05	2.6885E-06	4.666e-05 ± 2.689e-06	2	6.7%				
C_0122	ADMA	123831	HMDB0001539	203.1501	6.015118	14	100.0%	1.5981E-04	2.6034E-05	0.0001598 ± 2.603e-05	16	100.0%	1.6304E-04	2.0766E-05	0.000163 ± 2.077e-05	30	100.0%	9.8023E-01	-0.134	0.7136	0.8730
A_0112	ADP	6022	HMDB0001341	426.022	8.620229	11	78.6%	4.9350E-04	1.6678E-04	0.0004935 ± 0.0001668	16	100.0%	6.6128E-04	2.2260E-04	0.0006613 ± 0.0002226	27	90.0%	7.4628E-01	-0.863	0.0345	0.3224
C_0008	Ala	602	HMDB0000161,HMDB0001310	90.05484	7.054733	14	100.0%	1.2572E-01	3.0001E-02	0.1257 ± 0.03	16	100.0%	9.3853E-02	1.5153E-02	0.09385 ± 0.01515	30	100.0%	1.3395E+00	1.330	0.0020	0.0648
C_0049	Alloisoleucine	99288	HMDB0000557	132.1019	8.087611	14	100.0%	1.6162E-03	5.9763E-04	0.001616 ± 0.0005976	16	100.0%	1.7027E-03	3.7702E-04	0.001703 ± 0.000377	30	100.0%	9.4917E-01	-0.171	0.6454	0.8547
C_0109	Aminophylline	2153	HMDB0001889	181.0724	15.66211	6	42.9%	2.3923E-04	5.9152E-05	0.0002392 ± 5.915e-05	13	81.3%	3.1275E-04	2.8865E-04	0.0003128 ± 0.0002886	19	63.3%	7.6490E-01	-0.287	0.3941	0.7265
A_0101	AMP	6083	HMDB0000045	346.0557	7.535211	11	78.6%	2.2387E-04	5.0411E-05	0.0002239 ± 5.041e-05	15	93.8%	2.2499E-04	6.5092E-05	0.000225 ± 6.509e-05	26	86.7%	9.9499E-01	-0.018	0.9607	0.9812
C_0033	Anserine divalent	112072		121.0684	5.429151	1	7.1%	1.6265E-04		0.0001626	1	6.3%	8.5094E-05		8.509e-05	2	6.7%	1.9114E+00			
A_0046	Arabinonic acid	6602426	HMDB0000539	165.0397	7.016623	10	71.4%	1.3108E-04	2.4636E-05	0.0001311 ± 2.464e-05	12	75.0%	1.2375E-04	4.5329E-05	0.0001238 ± 4.533e-05	22	73.3%	1.0592E+00	0.188	0.6364	0.8543
C_0099	Arg	6322	HMDB0000517,HMDB0003416	175.119	5.613742	14	100.0%	2.7836E-02	7.5516E-03	0.02784 ± 0.007552	16	100.0%	2.1570E-02	5.4058E-03	0.02157 ± 0.005406	30	100.0%	1.2905E+00	0.085	0.0167	0.2335
C_0101	Argininic acid	160437	HMDB0003148	176.1025	7.122743	2	14.3%	7.8328E-05	1.2010E-07	7.833e-05 ± 1.201e-07	4	25.0%	6.2659E-05	1.9381E-05	6.266e-05 ± 1.938e-05	6	20.0%	1.2501E+00	0.747	0.2043	0.6257
A_0085	Ascorbate 2-sulfate	54676864		254.9814	10.83757	13	92.9%	4.4474E-04	1.2543E-04	0.0004447 ± 0.0001254	16	100.0%	3.7610E-04	1.4811E-04	0.0003761 ± 0.0001481	29	96.7%	1.1825E+00	0.482	0.1879	0.6139
C_0050	Asn	236	HMDB0000168,HMDB0033780	133.0606	8.175813	14	100.0%	8.9518E-03	2.9571E-03	0.008952 ± 0.002957	16	100.0%	7.3180E-03	9.8375E-04	0.007318 ± 0.0009837	30	100.0%	1.2233E+00	0.743	0.0665	0.4441
C_0053	Asp	424	HMDB0000191,HMDB0006483	134.0448	8.972937	14	100.0%	1.4430E-03	2.8880E-04	0.001443 ± 0.0002888	16	100.0%	1.6065E-03	5.5376E-04	0.001607 ± 0.0005538	30	100.0%	8.9821E-01	-0.353	0.3128	0.7091
A_0120	ATP	5957	HMDB0000538	505.988	9.206144	8	57.1%	2.2917E-04	3.9870E-05	0.0002292 ± 3.987e-05	12	75.0%	2.8171E-04	9.4277E-05	0.0002817 ± 9.428e-05	20	66.7%	8.1349E-01	-0.647	0.1059	0.4942
A_0063	Azelaic acid	2266	HMDB0000784	187.0971	9.563	5	35.7%	2.7857E-04	1.1686E-04	0.0002786 ± 0.0001169	3	18.8%	3.6129E-04	7.2349E-05	0.0003613 ± 7.235e-05	8	26.7%	7.7105E-01	-0.691	0.2632	0.6877
C_0029	Betaine	247	HMDB0000043	118.0862	8.708194	14	100.0%	3.1406E-02	5.0947E-03	0.03141 ± 0.005095	16	100.0%	2.6960E-02	6.2939E-03	0.02696 ± 0.006294	30	100.0%	1.1649E+00	0.723	0.0415	0.3463
C_0032	Betaine aldehyde_+H2O	249		120.102	5.873465	3	21.4%	4.1864E-05	1.5140E-05	4.186e-05 ± 1.514e-05	1	6.3%	6.2757E-05		6.276e-05	4	13.3%	6.6708E-01			
A_0004	Butyric acid Isobutyric acid	2646590	HMDB0000039 HMDB0001873	87.04496	8.01624	14	100.0%	5.5439E-04	2.0515E-04	0.0005544 ± 0.0002052	15	93.8%	5.1227E-04	1.0674E-04	0.0005123 ± 0.0001067	29	96.7%	1.0822E+00	0.253	0.5007	0.7877
C_0137	Butyrylcarnitine Isobutyrylcarnitine	439829168379	HMDB0002013 HMDB0000736	232.1543	7.393263	14	100.0%	2.9640E-04	9.9249E-05	0.0002964 ± 9.925e-05	16	100.0%	2.4876E-04	9.7992E-05	0.0002488 ± 9.799e-05	30	100.0%	1.1915E+00	0.470	0.1979	0.6257
C_0121	Caffeine	2519	HMDB0001847	195.0877	16.0454	13	92.9%	2.5116E-03	3.1390E-03	0.002512 ± 0.003139	16	100.0%	2.5427E-03	2.9356E-03	0.002543 ± 0.002936	29	96.7%	9.8775E-01	-0.010	0.9784	0.9845
A_0074	Capryloylglycine	84290	HMDB0000832	200.1292	6.416487	3	21.4%	2.1135E-04	1.3684E-04	0.0002113 ± 0.0001368	0	0.0%			N.A.	3	10.0%				
C_0128	Carboxymethyllysine	123800		205.1174	7.191907	2	14.3%	4.9457E-05	1.8434E-05	4.946e-05 ± 1.843e-05	0	0.0%			N.A.	2	6.7%				
C_0089	Carnitine	85	HMDB0000062	162.1126	6.660502	14	100.0%	2.9232E-02	8.5829E-03	0.02923 ± 0.008583	16	100.0%	2.7174E-02	5.0424E-03	0.02717 ± 0.005042	30	100.0%	1.0757E+00	0.290	0.4408	0.7472
A_0110	Cholic acid	221493	HMDB0000619	407.2804	5.779759	7	50.0%	5.9886E-04	7.0739E-04	0.0005989 ± 0.0007074	6	37.5%	2.8355E-04	8.2287E-05	0.0002835 ± 8.229e-05	13	43.3%	2.1120E+00	0.558	0.2850	0.7051
C_0018	Choline	305	HMDB0000097	104.1069	5.426113	14	100.0%	8.8070E-03	2.3275E-03	0.008807 ± 0.002327	16	100.0%	8.3669E-03	2.3641E-03	0.008367 ± 0.002364	30	100.0%	1.0526E+00	0.182	0.6120	0.8448
A_0056	cis-Aconitic acid	643757	HMDB0000072	173.0088	16.09065	14	100.0%	1.8802E-03	6.4809E-04	0.00188 ± 0.0006481	16	100.0%	1.9933E-03	3.3418E-04	0.001993 ± 0.0003342	30	100.0%	9.4326E-01	-0.218	0.5634	0.8180
A_0067	Citric acid	311	HMDB0000094	191.0195	15.93406	14	100.0%	2.0941E-02	5.8181E-03	0.02094 ± 0.005818	16	100.0%	2.1983E-02	2.9880E-03	0.02198 ± 0.002988	30	100.0%	9.5256E-01	-0.224	0.5527	0.8126
C_0102	Citrulline	9750	HMDB0000904	176.103	8.559241	14	100.0%	9.3209E-03	2.8392E-03	0.009321 ± 0.002839	16	100.0%	9.2541E-03	3.5164E-03	0.009254 ± 0.003516	30	100.0%	1.0072E+00	0.020	0.9545	0.9812
C_0046	Creatine	586	HMDB0000064	132.0767	6.911531	14	100.0%	1.9143E-02	1.4776E-02	0.01914 ± 0.01478	16	100.0%	1.2220E-02	7.2745E-03	0.01222 ± 0.007274	30	100.0%	1.5665E+00	0.591	0.1284	0.5135
C_0024	Creatinine	588	HMDB0000562	114.0661	5.749395	14	100.0%	2.6298E-02	4.8290E-03	0.0263 ± 0.004829	16	100.0%	2.8169E-02	6.6992E-03	0.02817 ± 0.006699	30	100.0%	9.3358E-01	-0.308	0.3840	0.7265
C_0067	Crotonic acid betaine	5280649	HMDB0250545	144.1026	6.481894	8	57.1%	6.3189E-05	2.2845E-05	6.319e-05 ± 2.285e-05	6	37.5%	5.5505E-05	2.8126E-05	5.551e-05 ± 2.813e-05	14	46.7%	1.1384E+00	0.286	0.5968	0.8442
C_0012	Cyclohexylamine	7965		100.112	6.016964	4	28.6%	1.0327E-04	5.5437E-05	0.0001033 ± 5.544e-05	12	75.0%	7.8777E-05	2.0300E-05	7.878e-05 ± 2.03e-05	16	53.3%	1.3109E+00	0.739	0.4460	0.7472
C_0134	Cystathionine	834	HMDB0000099	223.0745	7.717626	8	57.1%	8.9307E-05	4.5777E-05	8.931e-05 ± 4.578e-05	9	56.3%	6.7875E-05	3.7410E-05	6.788e-05 ± 3.741e-05	17	56.7%	1.3157E+00	0.490	0.3125	0.7091
C_0141	Cystine	595	HMDB0000192	241.0312	8.527994	14	100.0%	1.1775E-03	5.4214E-04	0.001178 ± 0.0005421	16	100.0%	1.3585E-03	5.2723E-04	0.001359 ± 0.0005272	30	100.0%	8.6675E-01	-0.330	0.3635	0.7265
C_0020	Diethanolamine	8113	HMDB0004437	106.0861	6.007692	13	92.9%	8.0072E-04	5.7799E-04	0.0008007 ± 0.000578	16	100.0%	8.5956E-04	6.0452E-04	0.0008596 ± 0.0006045	29	96.7%	9.3155E-01	-0.096	0.7915	0.9114
A_0051	Dihydroxyacetone phosphate	668	HMDB0001473	168.9902	9.862174	0	0.0%			N.A.	1	6.3%	1.9848E-04		0.0001985	1	3.3%				
C_0065	Ectoine	126041		143.0814	7.31579	5	35.7%	8.6897E-05	4.3637E-05	8.69e-05 ± 4.364e-05	2	12.5%	6.6867E-05	1.7575E-05	6.687e-05 ± 1.758e-05	7	23.3%	1.2996E+00	0.424	0.4280	0.7472

C_0003	Ethanolamine	700	HMDB0000149	62.05994	5.040129	14	100.0%	2.4048E-03	6.0022E-04	0.002405 ± 0.0006002	16	100.0%	2.5327E-03	5.6825E-04	0.002533 ± 0.0005682	30	100.0%	9.4950E-01	-0.213	0.5556	0.8126
A_0031	Ethanolamine phosphate	1015	HMDB0000224	140.0113	6.635341	4	28.6%	1.1683E-04	2.3261E-05	0.0001168 ± 2.326e-05	5	31.3%	1.4768E-04	5.9049E-05	0.0001477 ± 5.905e-05	9	30.0%	7.9111E-01	-0.581	0.3302	0.7141
C_0138	Ethylenediaminetriacetic acid	167782	HMDB0247201	235.0924	10.7165	14	100.0%	6.2232E-03	2.1878E-03	0.006223 ± 0.002188	16	100.0%	7.1279E-03	1.2491E-03	0.007128 ± 0.001249	30	100.0%	8.7308E-01	-0.503	0.1874	0.6139
A_0025	Ethylmalonic acid<br>Glutaric acid<br>Methylsuccinic acid	117567436950476	HMDB0000622<br>HMDB0000661<br>HMDB0001844	131.035	12.09067	2	14.3%	3.2555E-04	9.3165E-06	0.0003256 ± 9.317e-06	2	12.5%	3.2734E-04	7.8582E-05	0.0003273 ± 7.858e-05	4	13.3%	9.9452E-01	-0.018	0.9795	0.9845
C_0013	GABA	119	HMDB0000112	104.0705	6.042338	1	7.1%	1.5863E-04		0.0001586	0	0.0%			N.A.	1	3.3%				
C_0071	Gln	738	HMDB0000641,HMDB0003423	147.0764	8.346678	14	100.0%	1.0001E-01	3.3699E-02	0.1 ± 0.0337	16	100.0%	1.0342E-01	2.1892E-02	0.1034 ± 0.02189	30	100.0%	9.6707E-01	-0.118	0.7497	0.8875
C_0075	Glu	611	HMDB0000148,HMDB0003339	148.0604	8.493724	14	100.0%	4.0853E-02	2.1401E-02	0.04085 ± 0.0214	16	100.0%	3.7224E-02	2.4793E-02	0.03722 ± 0.02479	30	100.0%	1.0975E+00	0.152	0.6703	0.8561
A_0077	Glucaric acid	33037	HMDB0000663	209.0296	10.96603	2	14.3%	4.4671E-04	3.1152E-04	0.0004467 ± 0.0003115	0	0.0%			N.A.	2	6.7%				
A_0071	Gluconic acid	10690	HMDB0000625	195.0509	6.737862	14	100.0%	5.6480E-04	1.6710E-04	0.0005648 ± 0.0001671	16	100.0%	5.1380E-04	1.2832E-04	0.0005138 ± 0.0001283	30	100.0%	1.0993E+00	0.336	0.3628	0.7265
C_0104	Gluconolactone	7027	HMDB0000150	179.0553	17.00297	12	85.7%	2.8435E-04	8.0835E-05	0.0002844 ± 8.083e-05	11	68.8%	2.8822E-04	9.2333E-05	0.0002882 ± 9.233e-05	23	76.7%	9.8659E-01	-0.043	0.9163	0.9688
C_0106	Glucosamine	439213	HMDB0001514	180.0863	7.219753	13	92.9%	1.3025E-04	6.1890E-05	0.0001302 ± 6.189e-05	13	81.3%	9.4565E-05	3.0326E-05	9.456e-05 ± 3.033e-05	26	86.7%	1.3773E+00	0.709	0.0788	0.4441
A_0086	Glucose 1-phosphate	65533	HMDB0001586	259.0207	8.155578	0	0.0%			N.A.	1	6.3%	2.2746E-04		0.0002275	1	3.3%				
A_0069	Glucuronic acid	94715	HMDB0000127	193.035	6.707802	13	92.9%	3.0169E-04	1.3019E-04	0.0003017 ± 0.0001302	16	100.0%	2.5391E-04	6.1736E-05	0.0002539 ± 6.174e-05	29	96.7%	1.1882E+00	0.473	0.2410	0.6612
C_0088	Glutamic acid γ-methyl ester<br>2-Aminoadipic acid<br>O-Acetylhomoserine	6866292136439389	HMDB0061715<br>HMDB0000510	162.076	8.488172	14	100.0%	3.1477E-04	1.6724E-04	0.0003148 ± 0.0001672	16	100.0%	2.3467E-04	6.0555E-05	0.0002347 ± 6.056e-05	30	100.0%	1.3413E+00	0.637	0.1090	0.4970
C_0004	Gly	750	HMDB0000123	76.03921	6.550065	14	100.0%	4.7151E-02	1.2209E-02	0.04715 ± 0.01221	16	100.0%	3.7388E-02	5.7960E-03	0.03739 ± 0.005796	30	100.0%	1.2611E+00	1.001	0.0136	0.2051
C_0167	Gly-Pro-Hyp	11778669	HMDB0002171	286.1402	8.039211	0	0.0%			N.A.	5	31.3%	1.4690E-04	1.4055E-04	0.0001469 ± 0.0001406	5	16.7%				
A_0010	Glyceric acid	439194	HMDB0000139,HMDB0006372	105.0192	8.332778	14	100.0%	1.5293E-03	3.5477E-04	0.001529 ± 0.0003548	16	100.0%	1.4016E-03	2.8449E-04	0.001402 ± 0.0002845	30	100.0%	1.0912E+00	0.390	0.2914	0.7051
C_0011	Glycerol	753	HMDB0000131	93.05453	16.45462	14	100.0%	1.5472E-01	5.8021E-02	0.1547 ± 0.05802	16	100.0%	1.8907E-01	3.9759E-02	0.1891 ± 0.03976	30	100.0%	8.1832E-01	-0.681	0.0752	0.4441
C_0151	Glycerophosphocholine	439285	HMDB0000086	258.1102	16.16022	14	100.0%	5.0876E-03	6.7651E-03	0.005088 ± 0.006765	16	100.0%	3.2537E-03	1.0676E-03	0.003254 ± 0.001068	30	100.0%	1.5636E+00	0.382	0.3332	0.7141
C_0130	Glycerophosphorylethanolamine	444183	HMDB0000114	216.0635	16.13361	9	64.3%	1.9021E-04	9.7991E-05	0.0001902 ± 9.799e-05	7	43.8%	1.5545E-04	7.6979E-05	0.0001555 ± 7.698e-05	16	53.3%	1.2236E+00	0.367	0.4401	0.7472
A_0114	Glycocholic acid	10140	HMDB0000138	464.3025	5.670726	7	50.0%	2.0152E-04	5.2602E-05	0.0002015 ± 5.26e-05	1	6.3%	1.2076E-04		0.0001208	8	26.7%	1.6688E+00			
C_0072	Glycylsarcosine	93131	HMDB0252887	147.0765	6.781517	0	0.0%			N.A.	1	6.3%	8.0494E-05		8.049e-05	1	3.3%				
A_0001	Glyoxylic acid	760	HMDB0000119	72.99181	9.236948	1	7.1%	1.8560E-04		0.0001856	0	0.0%			N.A.	1	3.3%				
C_0100	Guanidinosuccinic acid	439918	HMDB0003157	176.0665	7.857009	12	85.7%	1.5535E-04	9.3219E-05	0.0001553 ± 9.322e-05	14	87.5%	1.0003E-04	3.4371E-05	0.0001 ± 3.437e-05	26	86.7%	1.5530E+00	0.788	0.0728	0.4441
C_0027	Guanidoacetic acid	763	HMDB0000128	118.0611	6.45934	14	100.0%	6.9779E-04	1.2667E-04	0.0006978 ± 0.0001267	16	100.0%	7.7348E-04	2.6608E-04	0.0007735 ± 0.0002661	30	100.0%	9.0214E-01	-0.346	0.3215	0.7141
C_0119	H-Asp(Gly-OH)-OH	99717	HMDB0011165	191.0662	9.249351	14	100.0%	2.1596E-04	6.9668E-05	0.000216 ± 6.967e-05	16	100.0%	1.9376E-04	7.2191E-05	0.0001938 ± 7.219e-05	30	100.0%	1.1146E+00	0.304	0.3993	0.7265
A_0014	Hexanoic acid<br>4-Methylpentanoic acid	88924275592	HMDB0000535<br>HMDB0000689	115.0764	7.328659	11	78.6%	1.9799E-04	8.4358E-05	0.000198 ± 8.436e-05	8	50.0%	2.6294E-04	1.8391E-04	0.0002629 ± 0.0001839	19	63.3%	7.5297E-01	-0.461	0.3761	0.7265
A_0058	Hippuric acid	464	HMDB0000714	178.0509	6.952924	12	85.7%	5.5271E-04	4.8915E-04	0.0005527 ± 0.0004892	15	93.8%	6.1801E-04	4.1907E-04	0.000618 ± 0.0004191	27	90.0%	8.9433E-01	-0.140	0.7171	0.8730
C_0083	His	773	HMDB0000177	156.0767	5.761272	14	100.0%	2.2837E-02	5.7151E-03	0.02284 ± 0.005715	16	100.0%	2.0772E-02	2.5187E-03	0.02077 ± 0.002519	30	100.0%	1.0994E+00	0.466	0.2279	0.6612
C_0116	Homoarginine	9085	HMDB0000670	189.1354	5.665762	14	100.0%	6.3545E-04	5.7863E-04	0.0006354 ± 0.0005786	16	100.0%	5.4992E-04	2.2040E-04	0.0005499 ± 0.0002204	30	100.0%	1.1555E+00	0.195	0.6094	0.8448
C_0142	Homocarnosine	10243361	HMDB0000745	241.1295	5.422153	1	7.1%	1.0204E-04		0.000102	0	0.0%			N.A.	1	3.3%				
C_0118	Homocitrulline	65072	HMDB0000679	190.1184	8.637722	13	92.9%	1.2871E-04	9.4645E-05	0.0001287 ± 9.464e-05	11	68.8%	1.0008E-04	4.7559E-05	0.0001001 ± 4.756e-05	24	80.0%	1.2860E+00	0.359	0.3510	0.7247
C_0031	Homoserine	12647	HMDB0000719	120.0655	7.846227	1	7.1%	1.2056E-04		0.0001206	3	18.8%	6.6238E-05	5.5522E-06	6.624e-05 ± 5.552e-06	4	13.3%	1.8201E+00			
A_0059	Homovanillic acid<br>Hydroxyphenyllactic acid<br>Veratric acid	173893787121	HMDB0000118<br>HMDB0000755<br>HMDB0059763	181.0501	6.831637	11	78.6%	1.9306E-04	3.9496E-05	0.0001931 ± 3.95e-05	12	75.0%	2.0624E-04	1.0261E-04	0.0002062 ± 0.0001026	23	76.7%	9.3611E-01	-0.161	0.6859	0.8673
C_0045	Hydroxyproline	5810	HMDB0000725	132.0655	9.272378	14	100.0%	3.9927E-03	2.1563E-03	0.003993 ± 0.002156	16	100.0%	4.1999E-03	1.9708E-03	0.0042 ± 0.001971	30	100.0%	9.5068E-01	-0.098	0.7868	0.9114
C_0021	Hypotaurine	107812	HMDB0000965	110.0269	13.48389	14	100.0%	2.2363E-04	7.6407E-05	0.0002236 ± 7.641e-05	16	100.0%	2.7139E-04	1.5301E-04	0.0002714 ± 0.000153	30	100.0%	8.2401E-01	-0.376	0.2823	0.7051
C_0056	Hypoxanthine	790	HMDB0000157	137.0457	8.796833	14	100.0%	1.6667E-03	6.3936E-04	0.001667 ± 0.0006394	16	100.0%	3.3969E-03	1.6486E-03	0.003397 ± 0.001649	30	100.0%	4.9066E-01	-1.251	0.0009	0.0615
C_0048	Ile	791	HMDB0000172	132.1018	7.900389	14	100.0%	6.3488E-02	2.0336E-02	0.06349 ± 0.02034	16	100.0%	5.0756E-02	7.9492E-03	0.05076 ± 0.007949	30	100.0%	1.2508E+00	0.824	0.0424	0.3463
C_0084	Imidazolelactic acid	793		157.0606	6.897761	14	100.0%	1.0306E-04	5.3655E-05	0.0001031 ± 5.365e-05	16	100.0%	9.3963E-05	4.1358E-05	9.396e-05 ± 4.136e-05	30	100.0%	1.0968E+00	0.186	0.6116	0.8448
A_0027	Iminodiacetic acid	8897	HMDB0011753	132.0301	7.695209	14	100.0%	1.2763E-03	4.3922E-04	0.001276 ± 0.0004392	14	87.5%	1.2362E-03	1.8658E-04	0.001236 ± 0.0001866	28	93.3%	1.0324E+00	0.115	0.7570	0.8875

A_0057	Indole-3-acetic acid	802	HMDB0000197	174.0558	7.051761	9	64.3%	2.0377E-04	6.3306E-05	0.0002038 ± 6.331e-05	9	56.3%	2.1206E-04	8.0713E-05	0.0002121 ± 8.071e-05	18	60.0%	9.6090E-01	-0.109	0.8116	0.9249
A_0076	Indole-3-lactic acid<br>5-Methoxyindoleacetic acid	67615718986	HMDB0000671<br>HMDB0004096	204.066	6.796609	7	50.0%	1.7137E-04	9.2636E-05	0.0001714 ± 9.264e-05	8	50.0%	1.7673E-04	8.8909E-05	0.0001767 ± 8.891e-05	15	50.0%	9.6967E-01	-0.056	0.9111	0.9688
A_0064	Indole-3-propionic acid	3744	HMDB0002302	188.0713	6.842966	3	21.4%	1.8793E-04	1.7188E-04	0.0001879 ± 0.0001719	5	31.3%	8.2917E-04	1.3007E-03	0.0008292 ± 0.001301	8	26.7%	2.2665E-01	-0.523	0.3352	0.7141
C_0158	Inosine	6021	HMDB0000195	269.088	14.73516	9	64.3%	1.9304E-04	5.1659E-05	0.000193 ± 5.166e-05	15	93.8%	3.2133E-04	2.0316E-04	0.0003213 ± 0.0002032	24	80.0%	6.0076E-01	-0.820	0.0329	0.3224
A_0020	Isethionic acid	7866	HMDB0003903	124.9911	9.111112	13	92.9%	2.8316E-04	6.4733E-05	0.0002832 ± 6.473e-05	16	100.0%	2.9100E-04	6.6441E-05	0.000291 ± 6.644e-05	29	96.7%	9.7305E-01	-0.116	0.7511	0.8875
C_0035	Isonicotinamide<br>Nicotinamide	15074936	HMDB0001406	123.0553	5.858789	6	42.9%	8.1752E-05	4.0140E-05	8.175e-05 ± 4.014e-05	14	87.5%	1.1189E-04	8.6491E-05	0.0001119 ± 8.649e-05	20	66.7%	7.3067E-01	-0.377	0.3018	0.7091
A_0006	Isovaleric acid<br>DL-2-Methylbutyric Acid<br>Valeric acid	1043083147991	HMDB0000718<br>HMDB0002176<br>HMDB0000892	101.0606	7.551194	13	92.9%	1.3199E-03	6.9989E-04	0.00132 ± 0.0006999	15	93.8%	7.8911E-04	3.7716E-04	0.0007891 ± 0.0003772	28	93.3%	1.6727E+00	0.937	0.0251	0.3031
A_0054	Isovalerylalanine<br>N-Acetylleucine<br>N-Acetylisoleucine<br>N-Hexanoylglycine	1292857091270362	HMDB0000747<br>HMDB0011756<br>HMDB0061684<br>HMDB0000701	172.0979	6.662387	5	35.7%	5.2136E-04	4.0180E-04	0.0005214 ± 0.0004018	0	0.0%			N.A.	5	16.7%				
C_0146	Isovalerylcarnitine	6426851	HMDB0000688	246.1701	7.590949	12	85.7%	1.5252E-04	5.6538E-05	0.0001525 ± 5.654e-05	15	93.8%	1.5103E-04	4.4103E-05	0.000151 ± 4.41e-05	27	90.0%	1.0099E+00	0.029	0.9410	0.9812
C_0129	Kynurenine	846	HMDB0000684	209.0921	7.801401	14	100.0%	5.4710E-04	1.2800E-04	0.0005471 ± 0.000128	16	100.0%	5.6912E-04	1.5428E-04	0.0005691 ± 0.0001543	30	100.0%	9.6131E-01	-0.150	0.6726	0.8561
A_0005	Lactic acid	612	HMDB0000190,HMDB0001311	89.02427	8.614199	14	100.0%	1.3242E-01	4.4255E-02	0.1324 ± 0.04426	16	100.0%	1.1571E-01	4.7907E-02	0.1157 ± 0.04791	30	100.0%	1.1444E+00	0.351	0.3295	0.7141
A_0073	Lauric acid	3893	HMDB0000638	199.1701	6.396886	13	92.9%	5.6730E-04	8.4276E-05	0.0005673 ± 8.428e-05	16	100.0%	5.4005E-04	7.9458E-05	0.00054 ± 7.946e-05	29	96.7%	1.0505E+00	0.324	0.3827	0.7265
C_0149	Lenticin	442106	HMDB0061115	247.1442	10.68646	6	42.9%	3.8603E-04	6.2123E-04	0.000386 ± 0.0006212	5	31.3%	2.7646E-04	2.3912E-04	0.0002765 ± 0.0002391	11	36.7%	1.3963E+00	0.205	0.7030	0.8730
C_0047	Leu	857	HMDB0000687	132.1017	7.987474	14	100.0%	1.1039E-01	2.9121E-02	0.1104 ± 0.02912	16	100.0%	9.4582E-02	1.3653E-02	0.09458 ± 0.01365	30	100.0%	1.1672E+00	0.692	0.0793	0.4441
C_0073	Lys	866	HMDB0000182,HMDB0003405	147.1129	5.43132	14	100.0%	5.4161E-02	1.4760E-02	0.05416 ± 0.01476	16	100.0%	4.3864E-02	6.0770E-03	0.04386 ± 0.006077	30	100.0%	1.2347E+00	0.922	0.0263	0.3031
A_0028	Malic acid	525	HMDB0000156,HMDB0000744	133.014	14.32516	14	100.0%	1.2429E-03	4.7881E-04	0.001243 ± 0.0004788	16	100.0%	1.2085E-03	4.4304E-04	0.001208 ± 0.000443	30	100.0%	1.0285E+00	0.073	0.8403	0.9411
A_0036	meso-Tartaric acid<br>Tartaric acid	447315444305	HMDB0000956	149.0102	14.66387	0	0.0%			N.A.	1	6.3%	3.7466E-04		0.0003747	1	3.3%				
C_0077	Met	876	HMDB0000696	150.0582	8.316165	14	100.0%	5.4789E-03	2.7518E-03	0.005479 ± 0.002752	16	100.0%	5.1648E-03	1.3639E-03	0.005165 ± 0.001364	30	100.0%	1.0608E+00	0.144	0.7028	0.8730
C_0092	Methionine sulfoxide	158980	HMDB0002005	166.0532	9.171844	14	100.0%	2.4365E-03	1.0269E-03	0.002436 ± 0.001027	16	100.0%	1.8004E-03	7.0441E-04	0.0018 ± 0.0007044	30	100.0%	1.3533E+00	0.712	0.0636	0.4441
C_0157	Metoprolol acid	62936	HMDB0254682	268.1538	8.152639	1	7.1%	2.8271E-04		0.0002827	0	0.0%			N.A.	1	3.3%				
A_0078	Mucic acid	3037582	HMDB0000639	209.0302	10.69363	13	92.9%	5.7959E-04	1.3211E-04	0.0005796 ± 0.0001321	15	93.8%	5.3854E-04	2.2369E-04	0.0005385 ± 0.0002237	28	93.3%	1.0762E+00	0.213	0.5542	0.8126
A_0080	Myristic acid	11005	HMDB0000806	227.2014	6.237223	13	92.9%	2.7784E-04	9.4057E-05	0.0002778 ± 9.406e-05	16	100.0%	3.3048E-04	1.2341E-04	0.0003305 ± 0.0001234	29	96.7%	8.4071E-01	-0.460	0.2036	0.6257
C_0016	N,N-Dimethylglycine	673	HMDB0000092	104.0705	8.480333	14	100.0%	1.7788E-03	6.3030E-04	0.001779 ± 0.0006303	16	100.0%	1.4535E-03	4.4884E-04	0.001454 ± 0.0004488	30	100.0%	1.2238E+00	0.585	0.1215	0.4973
C_0140	N-(1-Deoxy-1-fructosyl)glycine	131752250	HMDB0037848	238.0902	9.747446	1	7.1%	8.4755E-05		8.475e-05	0	0.0%			N.A.	1	3.3%				
C_0169	N-(1-Deoxy-1-fructosyl)leucine	131752244	HMDB0037840	294.1548	10.6081	13	92.9%	2.2451E-04	9.1680E-05	0.0002245 ± 9.168e-05	15	93.8%	1.1063E-04	3.0550E-05	0.0001106 ± 3.055e-05	28	93.3%	2.0293E+00	1.670	0.0007	0.0615
C_0163	N-(1-Deoxy-1-fructosyl)valine	131752247	HMDB0037844	280.1393	10.68193	13	92.9%	2.6544E-04	1.1284E-04	0.0002654 ± 0.0001128	15	93.8%	1.3933E-04	3.8559E-05	0.0001393 ± 3.856e-05	28	93.3%	1.9051E+00	1.498	0.0017	0.0648
A_0024	N-Acetylalanine<br>N-Acetyl-β-alanine<br>Propionylglycine	880647640698681	HMDB0000766<br>HMDB0000783	130.0509	7.281013	14	100.0%	3.4319E-04	6.9327E-05	0.0003432 ± 6.933e-05	16	100.0%	3.1199E-04	7.5956E-05	0.000312 ± 7.596e-05	30	100.0%	1.1000E+00	0.416	0.2496	0.6612
C_0159	N-Acetylcarnosine	9903482	HMDB0255058	269.1233	8.29688	3	21.4%	7.5727E-05	2.7181E-05	7.573e-05 ± 2.718e-05	4	25.0%	5.4356E-05	3.4529E-06	5.436e-05 ± 3.453e-06	7	23.3%	1.3932E+00	1.034	0.3058	0.7091
C_0133	N-Acetylgalactosamine<br>N-Acetylglucosamine<br>N-Acetylmannosamine	3571743917443928	HMDB0000853<br>HMDB0000215<br>HMDB0001129	222.0973	16.47506	14	100.0%	2.8184E-04	7.5627E-05	0.0002818 ± 7.563e-05	15	93.8%	3.2878E-04	8.1430E-05	0.0003288 ± 8.143e-05	29	96.7%	8.5723E-01	-0.580	0.1192	0.4973
A_0015	N-Acetylglycine	10972	HMDB0000532	116.0352	7.750618	10	71.4%	2.2732E-04	5.1053E-05	0.0002273 ± 5.105e-05	13	81.3%	2.5616E-04	9.1969E-05	0.0002562 ± 9.197e-05	23	76.7%	8.8743E-01	-0.360	0.3512	0.7247
C_0113	N-Acetyllysine	92907	HMDB0000446	189.1225	7.585693	0	0.0%			N.A.	1	6.3%	7.9363E-05		7.936e-05	1	3.3%				
C_0043	N-Acetylputrescine	122356	HMDB0002064	131.1177	6.603582	1	7.1%	1.0125E-04		0.0001013	6	37.5%	4.8730E-05	1.7765E-05	4.873e-05 ± 1.777e-05	7	23.3%	2.0778E+00			
C_0076	N-Acetylserine	65249	HMDB0002931	148.0604	17.07103	1	7.1%	2.2558E-04		0.0002256	0	0.0%			N.A.	1	3.3%				
A_0043	N-Acetylthreonine-1	152204	HMDB0062557	160.0613	6.806248	2	14.3%	1.6450E-04	5.6177E-05	0.0001645 ± 5.618e-05	0	0.0%			N.A.	2	6.7%				
A_0044	N-Acetylthreonine-2	152204	HMDB0062557	160.0614	6.936151	2	14.3%	1.4977E-04	1.6083E-05	0.0001498 ± 1.608e-05	1	6.3%	1.2834E-04		0.0001283	3	10.0%	1.1670E+00			
A_0040	N-Acetylvaline<br>Isovalerylglycine<br>N-Valerylglycine<br>2-Methylbutyrylglycine	6678954630447375	HMDB0011757<br>HMDB0000678<br>HMDB0000927<br>HMDB0000339	158.082	6.833899	3	21.4%	1.7479E-04	9.6428E-05	0.0001748 ± 9.643e-05	0	0.0%			N.A.	3	10.0%				
C_0042	N-Methylproline	557		130.0864	9.559014	12	85.7%	5.4783E-04	4.5737E-04	0.0005478 ± 0.0004574	8	50.0%	7.9059E-04	7.8095E-04	0.0007906 ± 0.0007809	20	66.7%	6.9293E-01	-0.385	0.4457	0.7472
C_0112	N1-Acetylspermidine	496	HMDB0001276	188.1759	5.006014	5	35.7%	4.1693E-05	2.6576E-05	4.169e-05 ± 2.658e-05	5	31.3%	3.9408E-05	8.9435E-06	3.941e-05 ± 8.943e-06	10	33.3%	1.0580E+00	0.104	0.8627	0.9499

C_0082	N1-Methyl-4-pyridone-5-carboxamide	440810	HMDB0004194	153.0655	13.95436	9	64.3%	1.7440E-04	9.7292E-05	0.0001744 ± 9.729e-05	13	81.3%	1.5018E-04	5.3167E-05	0.0001502 ± 5.317e-05	22	73.3%	1.1613E+00	0.315	0.5103	0.7938
A_0090	N2-Phenylacetylglutamine	92258	HMDB0006344	263.1034	6.297522	13	92.9%	5.5053E-04	3.7800E-04	0.0005505 ± 0.000378	15	93.8%	7.9490E-04	3.9126E-04	0.0007949 ± 0.0003913	28	93.3%	6.9258E-01	-0.616	0.1054	0.4942
C_0097	N5-Ethylglutamine	439378	HMDB0034365	175.1076	8.875207	11	78.6%	5.6287E-04	3.7079E-04	0.0005629 ± 0.0003708	4	25.0%	4.9230E-04	4.6092E-04	0.0004923 ± 0.0004609	15	50.0%	1.1433E+00	0.169	0.7951	0.9114
C_0117	N6,N6,N6-Trimethyllysine	440120	HMDB0001325	189.1586	5.66058	14	100.0%	3.4543E-04	1.3294E-04	0.0003454 ± 0.0001329	16	100.0%	3.4367E-04	8.1648E-05	0.0003437 ± 8.165e-05	30	100.0%	1.0051E+00	0.016	0.9661	0.9812
C_0114	N6-Acetyllysine	92832	HMDB0000206	189.1228	8.823788	13	92.9%	1.3322E-04	2.3766E-05	0.0001332 ± 2.377e-05	16	100.0%	1.1908E-04	1.6560E-05	0.0001191 ± 1.656e-05	29	96.7%	1.1187E+00	0.684	0.0839	0.4498
C_0098	N6-Formyllysine	70923		175.1091	8.524144	5	35.7%	8.5042E-05	2.9078E-05	8.504e-05 ± 2.908e-05	0	0.0%			N.A.	5	16.7%				
C_0086	N6-Methyllysine	164795	HMDB0002038	161.1284	5.595164	14	100.0%	1.4563E-03	1.0876E-03	0.001456 ± 0.001088	16	100.0%	9.0559E-04	6.7345E-04	0.0009056 ± 0.0006734	30	100.0%	1.6081E+00	0.602	0.1160	0.4973
A_0083	Nalidixic acid	4421		231.0772	6.350728	1	7.1%	3.0170E-03		0.003017	0	0.0%			N.A.	1	3.3%				
C_0115	Nω-Methylarginine	132862		189.1342	5.857226	2	14.3%	4.5223E-05	8.0705E-06	4.522e-05 ± 8.071e-06	6	37.5%	4.2868E-05	5.6873E-06	4.287e-05 ± 5.687e-06	8	26.7%	1.0549E+00	0.333	0.7537	0.8875
C_0125	O-Acetylcarnitine	439756	HMDB0000201	204.1231	7.048031	14	100.0%	6.9336E-03	2.1925E-03	0.006934 ± 0.002193	16	100.0%	8.5586E-03	2.5012E-03	0.008559 ± 0.002501	30	100.0%	8.1013E-01	-0.669	0.0683	0.4441
A_0030	o-Hydroxybenzoic acid	338	HMDB0001895	137.025	8.363089	4	28.6%	9.7150E-04	1.3700E-03	0.0009715 ± 0.00137	3	18.8%	4.3708E-03	6.8217E-03	0.004371 ± 0.006822	7	23.3%	2.2227E-01	-0.644	0.4802	0.7715
A_0070	o-Hydroxyhippuric acid	10253	HMDB0000840	194.0458	8.03016	0	0.0%			N.A.	1	6.3%	9.2294E-04		0.0009229	1	3.3%				
A_0075	o-Methoxyphenyl sulfate	22473	HMDB0060013	203.0027	7.802496	2	14.3%	2.2042E-04	4.7986E-05	0.0002204 ± 4.799e-05	2	12.5%	1.4519E-04	4.4337E-05	0.0001452 ± 4.434e-05	4	13.3%	1.5182E+00	0.931	0.2457	0.6612
A_0033	Octanoic acid-1<br>2-Ethylhexanoic acid	3798697	HMDB0000482<br>HMDB0031230	143.1075	6.806365	8	57.1%	1.8747E-02	2.7498E-02	0.01875 ± 0.0275	6	37.5%	1.5952E-04	1.6181E-05	0.0001595 ± 1.618e-05	14	46.7%	1.1752E+02	0.829	0.0975	0.4900
A_0032	Octanoic acid-2	379	HMDB0000482	143.1072	6.918369	5	35.7%	4.0114E-04	3.7736E-04	0.0004011 ± 0.0003774	3	18.8%	3.0058E-04	1.0257E-04	0.0003006 ± 0.0001026	8	26.7%	1.3346E+00	0.279	0.5987	0.8442
C_0168	Octanoylcarnitine	11953814	HMDB0000791	288.2169	8.078121	13	92.9%	1.5874E-04	1.1477E-04	0.0001587 ± 0.0001148	15	93.8%	4.4695E-04	7.4103E-04	0.0004469 ± 0.000741	28	93.3%	3.5516E-01	-0.509	0.1583	0.5642
C_0148	Octopine	440237		247.1399	8.319026	2	14.3%	9.0508E-05	2.7196E-05	9.051e-05 ± 2.72e-05	0	0.0%			N.A.	2	6.7%				
C_0051	Ornithine	389	HMDB0000214,HMDB0003374	133.0972	5.392298	14	100.0%	1.7177E-02	7.1646E-03	0.01718 ± 0.007165	16	100.0%	1.5181E-02	5.4484E-03	0.01518 ± 0.005448	30	100.0%	1.1315E+00	0.308	0.4040	0.7265
A_0093	p-Cresol glucuronide	154035	HMDB0011686	283.0843	6.177541	1	7.1%	8.2931E-05		8.293e-05	1	6.3%	1.5254E-04		0.0001525	2	6.7%	5.4367E-01			
A_0062	p-Cresol sulfate	4615423	HMDB0011635	187.0069	8.02889	14	100.0%	5.9844E-03	4.5439E-03	0.005984 ± 0.004544	16	100.0%	1.0148E-02	6.0008E-03	0.01015 ± 0.006001	30	100.0%	5.8973E-01	-0.754	0.0399	0.3463
A_0038	p-Hydroxyphenylacetic acid<br>3-Hydroxyphenylacetic acid	12712122	HMDB0000020<br>HMDB0000440	151.0412	7.135997	1	7.1%	2.5456E-04		0.0002546	1	6.3%	1.7345E-04		0.0001735	2	6.7%	1.4676E+00			
C_0107	Paraxanthine	4687	HMDB0001860	181.0717	15.12405	13	92.9%	8.8862E-04	6.2001E-04	0.0008886 ± 0.00062	16	100.0%	1.1528E-03	5.7082E-04	0.001153 ± 0.0005708	29	96.7%	7.7083E-01	-0.433	0.2483	0.6612
A_0048	Perillic acid	1256	HMDB0004586	165.0921	6.7479	3	21.4%	3.6990E-04	2.6191E-04	0.0003699 ± 0.0002619	8	50.0%	3.8809E-04	1.8189E-04	0.0003881 ± 0.0001819	11	36.7%	9.5313E-01	-0.082	0.9194	0.9688
C_0094	Phe	994	HMDB0000159	166.0862	8.565223	14	100.0%	4.2031E-02	1.0150E-02	0.04203 ± 0.01015	16	100.0%	3.9349E-02	5.9408E-03	0.03935 ± 0.005941	30	100.0%	1.0682E+00	0.319	0.3958	0.7265
A_0055	Phenol sulphate	74426	HMDB0060015	172.9919	8.609382	14	100.0%	2.3891E-03	1.4765E-03	0.002389 ± 0.001476	16	100.0%	2.2791E-03	1.6370E-03	0.002279 ± 0.001637	30	100.0%	1.0483E+00	0.068	0.8480	0.9444
C_0037	Phloroglucinol	359	HMDB0013675	127.0389	16.49015	14	100.0%	2.0945E-02	3.9682E-03	0.02094 ± 0.003968	16	100.0%	1.9202E-02	2.1001E-03	0.0192 ± 0.0021	30	100.0%	1.0907E+00	0.545	0.1571	0.5642
C_0111	Phosphorylcholine	1014	HMDB0001565	184.073	15.29224	13	92.9%	1.7256E-04	3.8676E-05	0.0001726 ± 3.868e-05	13	81.3%	1.6850E-04	1.0401E-04	0.0001685 ± 0.000104	26	86.7%	1.0241E+00	0.050	0.8969	0.9688
C_0041	Pipecolic acid	439227	HMDB0000070,HMDB0000716,HMDB0005960	130.0863	7.957697	14	100.0%	9.0547E-04	3.1578E-04	0.0009055 ± 0.0003158	16	100.0%	9.7671E-04	5.5141E-04	0.0009767 ± 0.0005514	30	100.0%	9.2707E-01	-0.152	0.6633	0.8561
C_0006	Piperidine	8082		86.09634	5.484082	0	0.0%			N.A.	1	6.3%	4.0626E-04		0.0004063	1	3.3%				
C_0026	Pro	614	HMDB0000162,HMDB0003411	116.0705	8.363788	14	100.0%	8.1632E-02	2.8624E-02	0.08163 ± 0.02862	16	100.0%	6.8685E-02	1.7726E-02	0.06868 ± 0.01773	30	100.0%	1.1885E+00	0.538	0.1578	0.5642
C_0136	Pro-Hyp	11902893	HMDB0006695	229.1184	7.640238	6	42.9%	2.6414E-04	1.4157E-04	0.0002641 ± 0.0001416	6	37.5%	2.6475E-04	1.4481E-04	0.0002648 ± 0.0001448	12	40.0%	9.9766E-01	-0.004	0.9942	0.9942
C_0131	Propionylcarnitine	188824	HMDB0000824	218.1386	7.254467	14	100.0%	5.0012E-04	1.9500E-04	0.0005001 ± 0.000195	16	100.0%	4.0399E-04	1.1442E-04	0.000404 ± 0.0001144	30	100.0%	1.2380E+00	0.596	0.1212	0.4973
A_0065	Pyrocatechol sulfate	3083879	HMDB0059724	188.9862	8.079215	14	100.0%	7.7991E-04	3.9012E-04	0.0007799 ± 0.0003901	16	100.0%	5.4953E-04	2.9872E-04	0.0005495 ± 0.0002987	30	100.0%	1.4192E+00	0.651	0.0849	0.4498
A_0003	Pyruvic acid	1060	HMDB0000243	87.00851	9.872863	0	0.0%			N.A.	1	6.3%	1.4627E-03		0.001463	1	3.3%				
A_0068	Quinic acid	6508	HMDB0003072	191.0564	6.814471	5	35.7%	2.4917E-04	1.3077E-04	0.0002492 ± 0.0001308	3	18.8%	3.0362E-04	2.1237E-04	0.0003036 ± 0.0002124	8	26.7%	8.2068E-01	-0.291	0.7159	0.8730
A_0081	Ribulose 5-phosphate	439184	HMDB0000618	229.0116	8.74326	10	71.4%	1.4548E-04	3.2323E-05	0.0001455 ± 3.232e-05	7	43.8%	1.5993E-04	3.9970E-05	0.0001599 ± 3.997e-05	17	56.7%	9.0966E-01	-0.385	0.4448	0.7472
C_0087	S-Allylcysteine	98280	HMDB0034323	162.0583	9.183522	7	50.0%	3.1630E-04	3.7297E-04	0.0003163 ± 0.000373	5	31.3%	2.2884E-04	1.2145E-04	0.0002288 ± 0.0001215	12	40.0%	1.3822E+00	0.270	0.5792	0.8348
C_0105	S-Carboxymethylcysteine	193653		180.0327	10.01038	1	7.1%	2.5640E-03		0.002564	0	0.0%			N.A.	1	3.3%				
C_0054	S-Methylcysteine	24417	HMDB0002108	136.0427	8.933099	14	100.0%	1.7626E-03	8.2303E-04	0.001763 ± 0.000823	16	100.0%	1.5720E-03	6.4654E-04	0.001572 ± 0.0006465	30	100.0%	1.1212E+00	0.253	0.4916	0.7834
C_0078	S-Methylcysteine-S-oxide	82142	HMDB0029432	152.0376	10.52039	14	100.0%	1.2487E-03	8.6577E-04	0.001249 ± 0.0008658	16	100.0%	7.7797E-04	5.1810E-04	0.000778 ± 0.0005181	30	100.0%	1.6051E+00	0.653	0.0906	0.4673
C_0091	S-Methylmethionine	145692		164.0744	5.773442	3	21.4%	5.1525E-05	3.2274E-05	5.153e-05 ± 3.227e-05	2	12.5%	6.2299E-05	1.0064E-05	6.23e-05 ± 1.006e-05	5	16.7%	8.2707E-01	-0.290	0.6331	0.8543
C_0009	Sarcosine	1088	HMDB0000271	90.05485	7.404779	14	100.0%	1.0581E-03	2.9834E-04	0.001058 ± 0.0002983	16	100.0%	7.7550E-04	2.4173E-04	0.0007755 ± 0.0002417	30	100.0%	1.3644E+00	0.975	0.0092	0.1817

C_0123	SDMA	169148	HMDB0003334	203.1503	6.112123	14	100.0%	2.2862E-04	8.9524E-05	0.0002286 ± 8.952e-05	16	100.0%	2.3753E-04	5.5811E-05	0.0002375 ± 5.581e-05	30	100.0%	9.6250E-01	-0.118	0.7509	0.8875
C_0019	Ser	617	HMDB0000187,HMDB0003406	106.0497	7.799534	14	100.0%	2.1376E-02	5.0146E-03	0.02138 ± 0.005015	16	100.0%	1.9658E-02	3.4124E-03	0.01966 ± 0.003412	30	100.0%	1.0874E+00	0.395	0.2909	0.7051
C_0103	Serotonin	5202	HMDB0000259	177.1022	6.839229	7	50.0%	1.1605E-04	4.9698E-05	0.000116 ± 4.97e-05	13	81.3%	9.8329E-05	4.3614E-05	9.833e-05 ± 4.361e-05	20	66.7%	1.1802E+00	0.371	0.4444	0.7472
C_0066	Stachydrine	115244	HMDB0004827	144.1019	8.848198	14	100.0%	7.1064E-03	7.5282E-03	0.007106 ± 0.007528	16	100.0%	3.5060E-03	4.5520E-03	0.003506 ± 0.004552	30	100.0%	2.0269E+00	0.573	0.1344	0.5269
A_0016	Succinic acid	1110	HMDB0000254	117.0191	14.13567	14	100.0%	1.1992E-03	6.8877E-04	0.001199 ± 0.0006888	16	100.0%	9.5263E-04	3.6215E-04	0.0009526 ± 0.0003621	30	100.0%	1.2588E+00	0.445	0.2441	0.6612
A_0089	Sulfotyrosine	514186		260.0218	6.712453	0	0.0%			N.A.	1	6.3%	2.1012E-04		0.0002101	1	3.3%				
C_0036	Taurine	1123	HMDB0000251	126.0219	16.45911	14	100.0%	2.2276E-03	5.7467E-04	0.002228 ± 0.0005747	16	100.0%	2.3052E-03	5.7532E-04	0.002305 ± 0.0005753	30	100.0%	9.6634E-01	-0.131	0.7152	0.8730
A_0045	Terephthalic acid	7489	HMDB0002428	165.0191	11.99497	14	100.0%	2.7495E-04	5.8935E-05	0.0002749 ± 5.893e-05	16	100.0%	2.2683E-04	3.0630E-05	0.0002268 ± 3.063e-05	30	100.0%	1.2121E+00	1.018	0.0128	0.2051
C_0108	Theobromine	5429	HMDB0002825	181.0722	16.14325	12	85.7%	5.8387E-04	3.7998E-04	0.0005839 ± 0.00038	14	87.5%	4.5840E-04	1.8930E-04	0.0004584 ± 0.0001893	26	86.7%	1.2737E+00	0.415	0.3148	0.7091
C_0155	Thiamine	1130	HMDB0000235	265.1114	5.206088	2	14.3%	1.3375E-04	8.7016E-06	0.0001338 ± 8.702e-06	0	0.0%			N.A.	2	6.7%				
C_0052	Thiaproline	9934		134.0273	10.70381	7	50.0%	8.2978E-05	2.2682E-05	8.298e-05 ± 2.268e-05	13	81.3%	8.8228E-05	2.4859E-05	8.823e-05 ± 2.486e-05	20	66.7%	9.4050E-01	-0.208	0.6409	0.8545
A_0035	Thiodiglycolic acid	31277	HMDB0042032	148.9917	13.85483	1	7.1%	9.0066E-04		0.0009007	0	0.0%			N.A.	1	3.3%				
C_0030	Thr	6288	HMDB0000167	120.0653	8.173962	14	100.0%	4.4092E-02	2.2918E-02	0.04409 ± 0.02292	16	100.0%	3.8569E-02	7.6086E-03	0.03857 ± 0.007609	30	100.0%	1.1432E+00	0.324	0.4023	0.7265
A_0029	Threonic acid	5460407	HMDB0000943	135.0298	7.535931	14	100.0%	2.1280E-03	4.5541E-04	0.002128 ± 0.0004554	16	100.0%	1.9270E-03	4.6946E-04	0.001927 ± 0.0004695	30	100.0%	1.1043E+00	0.422	0.2447	0.6612
C_0058	Trigonelline	5570	HMDB0000875	138.0549	8.127747	13	92.9%	2.8859E-04	1.7371E-04	0.0002886 ± 0.0001737	16	100.0%	3.9453E-04	4.3151E-04	0.0003945 ± 0.0004315	29	96.7%	7.3149E-01	-0.301	0.3803	0.7265
C_0001	Trimethylamine	1146	HMDB0000906	60.08038	4.687942	1	7.1%	1.4510E-04		0.0001451	1	6.3%	2.8898E-04		0.000289	2	6.7%	5.0212E-01			
C_0005	Trimethylamine N-oxide	1145	HMDB0000925	76.07556	5.233297	14	100.0%	4.9866E-03	5.4967E-03	0.004987 ± 0.005497	16	100.0%	3.9498E-03	2.6186E-03	0.00395 ± 0.002619	30	100.0%	1.2625E+00	0.240	0.5273	0.8025
C_0127	Trp	1148	HMDB0000929	205.0972	8.519042	14	100.0%	1.6698E-02	5.2127E-03	0.0167 ± 0.005213	16	100.0%	1.4779E-02	1.9769E-03	0.01478 ± 0.001977	30	100.0%	1.1298E+00	0.487	0.2123	0.6403
C_0110	Tyr	1153	HMDB0000158	182.0811	8.765916	14	100.0%	2.1496E-02	6.1910E-03	0.0215 ± 0.006191	16	100.0%	2.0273E-02	3.7876E-03	0.02027 ± 0.003788	30	100.0%	1.0603E+00	0.236	0.5282	0.8025
C_0023	Uracil	1174	HMDB0000300	113.0345	16.49347	5	35.7%	1.4059E-04	1.7562E-05	0.0001406 ± 1.756e-05	8	50.0%	1.3843E-04	2.5800E-05	0.0001384 ± 2.58e-05	13	43.3%	1.0156E+00	0.087	0.8610	0.9499
C_0002	Urea	1176	HMDB0000294	61.03957	15.84262	14	100.0%	7.1591E-01	2.3526E-01	0.7159 ± 0.2353	16	100.0%	6.5207E-01	1.3175E-01	0.6521 ± 0.1317	30	100.0%	1.0979E+00	0.332	0.3793	0.7265
A_0050	Uric acid	1175	HMDB0000289	167.0206	7.28616	14	100.0%	2.4284E-02	6.0953E-03	0.02428 ± 0.006095	16	100.0%	2.5193E-02	3.8094E-03	0.02519 ± 0.003809	30	100.0%	9.6393E-01	-0.177	0.6351	0.8543
C_0145	Uridine	6029	HMDB0000296	245.077	16.49658	14	100.0%	1.3191E-03	2.8466E-04	0.001319 ± 0.0002847	16	100.0%	1.4611E-03	2.7921E-04	0.001461 ± 0.0002792	30	100.0%	9.0286E-01	-0.490	0.1804	0.6139
C_0062	Urocanic acid	736715	HMDB0000301	139.0496	6.476333	0	0.0%			N.A.	3	18.8%	8.6128E-05	3.0852E-06	8.613e-05 ± 3.085e-06	3	10.0%				
C_0028	Val	1182	HMDB0000883	118.0861	7.759718	14	100.0%	1.4794E-01	2.8741E-02	0.1479 ± 0.02874	16	100.0%	1.2779E-01	1.5357E-02	0.1278 ± 0.01536	30	100.0%	1.1577E+00	0.087	0.0298	0.3196
C_0081	Xanthine	1188	HMDB0000292	153.0408	14.7549	14	100.0%	4.3466E-04	5.2057E-04	0.0004347 ± 0.0005206	16	100.0%	3.7124E-04	1.5589E-04	0.0003712 ± 0.0001559	30	100.0%	1.1708E+00	0.166	0.6669	0.8561
C_0010	β-Ala	239	HMDB0000056	90.05495	5.791746	14	100.0%	6.9159E-04	4.2129E-04	0.0006916 ± 0.0004213	16	100.0%	5.8266E-04	2.6031E-04	0.0005827 ± 0.0002603	30	100.0%	1.1870E+00	0.308	0.4116	0.7335
A_0019	β-Hydroxyisovaleric acid-1<br>2-Hydroxyvaleric acid-1<br>2-Hydroxyisovaleric acid-1	693629800999823	HMDB0000754<br>HMDB0001863<br>HMDB0000407	117.0556	7.48272	14	100.0%	3.4909E-04	1.2956E-04	0.0003491 ± 0.0001296	16	100.0%	2.8476E-04	1.0056E-04	0.0002848 ± 0.0001006	30	100.0%	1.2259E+00	0.545	0.1455	0.5593
A_0018	β-Hydroxyisovaleric acid-2<br>2-Hydroxyvaleric acid-2<br>2-Hydroxyisovaleric acid-2	693629800999823	HMDB0000754<br>HMDB0001863<br>HMDB0000407	117.0556	7.562933	14	100.0%	2.2514E-03	3.9591E-03	0.002251 ± 0.003959	16	100.0%	1.5864E-03	8.5096E-04	0.001586 ± 0.000851	30	100.0%	1.4192E+00	0.234	0.5477	0.8126
C_0069	γ-Butyrobetaine	134	HMDB0001161	146.1177	6.351908	14	100.0%	8.2271E-04	2.1251E-04	0.0008227 ± 0.0002125	16	100.0%	9.7886E-04	2.2201E-04	0.0009789 ± 0.000222	30	100.0%	8.4048E-01	-0.698	0.0593	0.4307
C_0120	γ-Carboxyglutamic acid	40772	HMDB0041900	192.0506	11.00591	1	7.1%	6.8484E-05		6.848e-05	2	12.5%	7.2551E-05	1.2535E-05	7.255e-05 ± 1.253e-05	3	10.0%	9.4394E-01			
C_0132	γ-Glu-Ala	440103	HMDB0006248	219.0975	9.473926	14	100.0%	6.6898E-04	2.8547E-04	0.000669 ± 0.0002855	16	100.0%	4.2617E-04	1.2781E-04	0.0004262 ± 0.0001278	30	100.0%	1.5697E+00	1.095	0.0090	0.1817
C_0080	γ-Glu-Arg_divalent	20719180		152.5841	6.805906	14	100.0%	4.0321E-04	9.5963E-05	0.0004032 ± 9.596e-05	16	100.0%	3.2608E-04	1.0948E-04	0.0003261 ± 0.0001095	30	100.0%	1.2365E+00	0.726	0.0492	0.3857
C_0153	γ-Glu-Asn	131801686	HMDB0029144	262.103	9.79623	8	57.1%	9.6532E-05	1.6884E-05	9.653e-05 ± 1.688e-05	8	50.0%	7.0033E-05	1.1959E-05	7.003e-05 ± 1.196e-05	16	53.3%	1.3784E+00	1.712	0.0032	0.0908
C_0171	γ-Glu-Citrulline			305.146	10.12013	7	50.0%	9.1879E-05	1.8892E-05	9.188e-05 ± 1.889e-05	6	37.5%	9.3389E-05	2.4349E-05	9.339e-05 ± 2.435e-05	13	43.3%	9.8384E-01	-0.065	0.9044	0.9688
C_0160	γ-Glu-Gln	150914	HMDB0011738	276.1189	9.977338	14	100.0%	1.9935E-03	8.6733E-04	0.001993 ± 0.0008673	16	100.0%	1.8695E-03	6.7948E-04	0.001869 ± 0.0006795	30	100.0%	1.0663E+00	0.156	0.6700	0.8561
C_0161	γ-Glu-Glu	92865	HMDB0011737	277.103	10.05336	14	100.0%	3.2452E-04	1.3259E-04	0.0003245 ± 0.0001326	16	100.0%	2.9633E-04	1.1500E-04	0.0002963 ± 0.000115	30	100.0%	1.0951E+00	0.222	0.5421	0.8126
C_0126	γ-Glu-Gly	165527	HMDB0011667	205.0819	9.286927	14	100.0%	1.3005E-03	6.6972E-04	0.0013 ± 0.0006697	16	100.0%	8.4381E-04	3.0848E-04	0.0008438 ± 0.0003085	30	100.0%	1.5412E+00	0.873	0.0310	0.3196
C_0166	γ-Glu-His	7017195	HMDB0029151	285.1191	6.807603	14	100.0%	8.8826E-05	2.5834E-05	8.883e-05 ± 2.583e-05	15	93.8%	8.1601E-05	2.5869E-05	8.16e-05 ± 2.587e-05	29	96.7%	1.0885E+00	0.272	0.4586	0.7553
C_0152	γ-Glu-Ile<br>γ-Glu-Leu	22885096151023	HMDB0011170<br>HMDB0011171	261.1445	9.903552	14	100.0%	5.1727E-04	1.8015E-04	0.0005173 ± 0.0001802	16	100.0%	3.7622E-04	1.0668E-04	0.0003762 ± 0.0001067	30	100.0%	1.3749E+00	0.943	0.0183	0.2396

C_0061	γ-Glu-Lys_divalent	65254		138.581	6.705145	14	100.0%	1.0130E-03	3.0029E-04	0.001013 ± 0.0003003	16	100.0%	7.5655E-04	2.0811E-04	0.0007566 ± 0.0002081	30	100.0%	1.3390E+00	0.978	0.0134	0.2051
C_0162	γ-Glu-Met	7009567	HMDB0034367	279.101	9.940585	8	57.1%	9.1507E-05	1.8141E-05	9.151e-05 ± 1.814e-05	6	37.5%	7.8258E-05	2.2787E-05	7.826e-05 ± 2.279e-05	14	46.7%	1.1693E+00	0.614	0.2699	0.6962
C_0154	γ-Glu-Ornitine H-Asp(Lys-OH)-OH	189156165361	HMDB0002248	262.1396	6.638941	14	100.0%	1.1483E-04	3.1147E-05	0.0001148 ± 3.115e-05	16	100.0%	9.7535E-05	3.8039E-05	9.754e-05 ± 3.804e-05	30	100.0%	1.1774E+00	0.481	0.1820	0.6139
C_0170	γ-Glu-Phe	111299	HMDB0000594	295.1285	9.996674	10	71.4%	8.7847E-05	1.5457E-05	8.785e-05 ± 1.546e-05	11	68.8%	7.9922E-05	1.7950E-05	7.992e-05 ± 1.795e-05	21	70.0%	1.0992E+00	0.452	0.2908	0.7051
C_0139	γ-Glu-Ser	22844748	HMDB0029158	235.0926	9.686337	14	100.0%	2.6720E-04	8.5233E-05	0.0002672 ± 8.523e-05	16	100.0%	2.1205E-04	5.6700E-05	0.0002121 ± 5.67e-05	30	100.0%	1.2601E+00	0.752	0.0518	0.3907
A_0084	γ-Glu-Taurine	68759	HMDB0004195	253.0499	6.695853	1	7.1%	1.4126E-04		0.0001413	0	0.0%			N.A.	1	3.3%				
C_0150	γ-Glu-Thr	53861142	HMDB0029159	249.1081	9.771067	14	100.0%	1.6563E-04	7.1881E-05	0.0001656 ± 7.188e-05	15	93.8%	1.3485E-04	4.2491E-05	0.0001349 ± 4.249e-05	29	96.7%	1.2282E+00	0.511	0.1788	0.6139
C_0174	γ-Glu-Tyr	94340	HMDB0011741	311.1233	10.14523	4	28.6%	7.9501E-05	1.8648E-05	7.95e-05 ± 1.865e-05	4	25.0%	8.1677E-05	3.2447E-05	8.168e-05 ± 3.245e-05	8	26.7%	9.7336E-01	-0.071	0.9122	0.9688
C_0147	γ-Glu-Val	7015683	HMDB0011172	247.1288	9.755984	14	100.0%	5.6717E-04	1.7684E-04	0.0005672 ± 0.0001768	16	100.0%	4.0632E-04	1.2485E-04	0.0004063 ± 0.0001248	30	100.0%	1.3959E+00	1.035	0.0093	0.1817

Peak ID consists of the initial letter of the measurement mode followed by a sequential number; C denotes cation mode and A denotes anion mode. Metabolite name indicates the candidate compound obtained by matching the detected peak's m/z and migration time (MT) to the HMT in-house database/library. Metabolites are listed when a corresponding peak was detected in at least one sample; metabolites with no detected peak across all samples were not reported in the quantitative output. Values are Relative Area (normalized peak area; arbitrary units) and are presented as mean ± SD using detected values only. N.D., not detected (below the limit of detection); N.A., not available (could not be computed due to insufficient data). n_CO and n_CON denote the number of samples with detected values used to compute mean and SD; detection rate (%) is calculated as n_group divided by the total number of samples in that group (CO: 14; CON: 16). Mean ratio (CO/CON) was calculated using the CON mean as the denominator. Welch p values were obtained by two-sided Welch's t-test; p was set to N.A. when either group had <2 detected values. BH-FDR q values were calculated using the Benjamini–Hochberg procedure across metabolites with available p values in this table. Hedges' g (standardized mean difference; small-sample corrected) was calculated as (mean_CO − mean_CON)/pooled SD; positive values indicate higher levels in the CO group. BH-FDR q values were calculated … across metabolites with available p values (n = 196) in this table.
