# Supplementary material for: Sixty Years After a Coal Mine Disaster: Serum Metabolomic Profiles in Older Adults with Long-Term Sequelae of Carbon Monoxide Poisoning: A Cross-Sectional Study
Source: Metabolites. 2026 Feb 12;16(2):126. doi: 10.3390/metabo16020126 (PMC12943369; doi:10.3390/metabo16020126)
Supplement: Supplementary file 1 [file metabolites-16-00126-s001.zip › metabolites-4124646-supplementary/Revised Supplementary files/S1.pdf]

Supplementary Table S1. ANCOVA/linear regression for serum BDNF adjusted for age and MMSE

Outcome	Unit	Model	Covariates	Group contrast	CO (n, mean±SD)	CON (n, mean±SD)	Adjusted mean difference (CON − CO)	95% CI	Standardized β	p-value
Serum BDNF	pg/mL	ANCOVA / linear regression	Age, MMSE	CON vs CO	14, 18373.6 ± 8130.7	16, 23945.6 ± 8206.0	4,119.8	553.3 to 7,686.2	0.4912	0.0252

Adjusted mean difference and 95% CI are from the age- and MMSE-adjusted model and are expressed as CON − CO.
