# Supplementary material for: Sixty Years After a Coal Mine Disaster: Serum Metabolomic Profiles in Older Adults with Long-Term Sequelae of Carbon Monoxide Poisoning: A Cross-Sectional Study
Source: Metabolites. 2026 Feb 12;16(2):126. doi: 10.3390/metabo16020126 (PMC12943369; doi:10.3390/metabo16020126)
Supplement: Supplementary file 1 [file metabolites-16-00126-s001.zip › metabolites-4124646-supplementary/Revised Supplementary files/S6.pdf]

Supplementary Table S6. Targeted metabolite panel (n = 1,183): annotated metabolite list.

Column 1	Column 2	Column 3	Column 4	Column 5	Column 6	Column 7	Column 8	Column 9	Column 10
(Aminomethyl)phosphonic acid	3-(4-Hydroxy-3-methoxyphenyl)pro	5-Methylfuran-2-carboxylic acid	Bis(p-nitrophenyl)phosphate	Ethanolamine	Histamine	Metaraminol	N-Methylanthranilic acid	Phloretin	Suberic acid
(Methylthio)acetic acid	3-(4-Hydroxyphenyl)propionic acid	5-Methyltetrahydrofolic acid	Buformin	Ethanolamine phosphate	Histidinol	Methanesulfonic acid	N-Methylaspartic acid	Phloroglucinol	Succinic acid
1,2,3,4-Tetrahydroisoquinoline	3-(Methylthio)propionic acid	5-Oxo-2-tetrahydrofurancarboxylic	Butyric acid	Ethionine	HMBPP	Methionine sulfoxide	N-Methylglutamic acid	Phosphocreatine	Succinic semialdehyde
1,2,3-Propanetricarboxylic acid	3-Acetylacrylic acid	5-Oxohexanoic acid	Butyrylcarnitine	Ethyl glucuronide	HMG CoA	Methionine sulfoximine	N-Methylnicotinamide	Phosphoenolpyruvic acid	Succinyl CoA8
1,3-Diaminopropane	3-Amino-1,2,4-triazole	5-Oxoproline	Cadaverine	Ethylacetimidate	Homoarginine	Methotrimeprazine	N-Methylphenylethanolamine	Phosphoglycolic acid	Succinylacetone
1,3-Phenylenediamine	3-Amino-2-piperidone	5-Sulfosalicylic acid	Caffeic acid	Ethylenediaminetriacetic acid	Homocarnosine	Methoxamine	N-Methylproline	Phosphomevalonic acid	Succinyladenosine
1-Aminocyclopentanecarboxylic acid	3-Amino-5-hydroxypyrazole	6,8-Thioctic acid	Caffeine	Ethylmalonic acid	Homocitric acid	Methyl aminolevulinate	N-Methylputrescine	Phosphonoacetic acid	Succinylcarnitine
1-Aminocyclopropane-1-carboxylic a	3-Aminobutyric acid	6-Aminohexanoic acid	cAMP	Etidronic acid	Homocitrulline	Methyl sulfate	N-Methylserotonin	Phosphoramidon	Succinylcholine
1-Aminoethylphosphonic acid	3-Aminoisobutyric acid	6-Hydroxydopamine	Canavanine	FAD	Homocysteic acid	Methylguanidine	N-Methyltryptamine	Phosphorylcholine	Sucrose 6'-phosphate
1-Deoxynojirimycin	3-Aminopropane-1,2-diol	6-Hydroxyhexanoic acid	Capryloylglycine	Farnesyl pyrophosphate	Homocysteine	Methylmalonic acid	N-Methyltryptophan	Phthalic acid	Sulfanilamide
1-Methyl-2-pyrrolidone	3-Aminopropionitrile	6-Hydroxynicotinic acid	Carbachol	Ferulic acid	Homocysteinesulfinic acid	Methylmalonyl CoA8	N-Methyltyramine	Phytic acid	Sulfanilic acid
1-Methyl-4-imidazoleacetic acid	3-Chloroalanine	6-Mercaptopurine	Carbamoylphosphate	FMN	Homocysteinethiolactone	Methylsuccinic acid	N-Nitrosodiethanolamine	Picolinamide	Sulfathiazole
1-Methyl-4-phenyl-1,2,3,6-tetrahyd	3-Chlorotyrosine	6-Phosphogluconic acid	Carbendazim	Folic acid	Homocystine	Methysergide	N-Propionylalanine	Picolinic acid	Sulfotyrosine
1-Methyladenine	3-Cresotinic acid	7,8-Dihydrobiopterin	Carboxymethyllysine	Formiminoglutamic acid	Homogentisic acid	Metoprolol acid	N-Succinyl-5-aminoimidazole-4-carb	Pilocarpine	Synephrine
1-Methyladenosine	3-Dehydroshikimic acid	7,8-Dihydrofolic acid	Carnitine	Fructose 1,6-diphosphate	Homoserine	Mevalolactone	N-Valerylglycine	Pimelic acid	Syringic acid
1-Methylhistamine	3-Deoxy-D-manno-2-octulosonic aci	7,8-Dihydroneopterin	Carnosine	Fructose 6-phosphate	Homoserinelactone	Mevalonic acid	NAADP	Pipecolic acid	Tacrine
1-Methylhistidine	3-Fluoro-4-hydroxybenzoic acid	7-Amino-4-hydroxy-2-naphthalenes	Castanospermine	Fumaric acid	Homovanillic acid	Mimosine	NAD+	Piperazine	Tartaric acid
1-Methylhydantoin	3-Furoic acid	7-Methylguanine	cCMP2 2',3'-cCMP2	Fumaric acid monomethyl ester	Hordenine	Minocycline	NADH	Piperidine	Taurine
1-Methylnicotinamide	3-Guanidinopropionic acid	7-Methylxanthine	CDP	Furosemide	Hydroxyatrazine	Monoethyl malonic acid	NADP+	Porphobilinogen	Taurocholic acid
1-Methyluric acid	3-Hydroxy-2-methyl-4-pyrone	8-Anilino-1-naphthalenesulfonic aci	CDP-choline	GABA	Hydroxycitric acid	Monoethyl phosphate	NADPH	ppGpp	Taurocyamine
1-Phenylethylamine	3-Hydroxy-3-methylglutaric acid	8-Hydroxy-2'-deoxyguanosine	CDP-ethanolamine	Galactosamine	Hydroxyindole	Monomethyl glutaric acid	Nalidixic acid	Pralidoxime	Terbutaline
10-Hydroxydecanoic acid	3-Hydroxyanthranilic acid	8-Hydroxyadenine	cGMP	Galacturonate 1-phosphate	Hydroxyphenyllactic acid	Morpholine	Neamine	Prazosin	Terephthalic acid
11-Aminoundecanoic acid	3-Hydroxybutyric acid	8-Hydroxyoctanoic acid	Chelidonic acid	Galacturonic acid	Hydroxyproline	Mucic acid	Neomycin B	Pristanic acid	Tetrahydrofolic acid
15-Methyl prostaglandin F2α	3-Hydroxydecanoic acid	Abscisic acid	Chloramphenicol	Gallic acid	Hydroxyurea	Muramic acid	Neopterin	Pro	Tetrahydropalmatine
1H-Imidazole-4-propionic acid	3-Hydroxyhexanoic acid	Acetanilide	Chloroacetic acid	γ-Butyrobetaine	Hypotaurine	Muscimol	Neostigmine	Pro-Hyp	Tetrahydrouridine
2'-CMP3 3'-CMP3	3-Hydroxyisobutyric acid	Acetoacetamide	Cholic acid	γ-Carboxyglutamic acid	Hypoxanthine	myo-Inositol 1-phosphate6	Nicotinamide	Procaine	Theobromine
2'-Deoxyadenosine4 5'-Deoxyadenos	3-Hydroxyisovalerylcarnitine	Acetoacetic acid	Choline	γ-Glu-Ala	Ibotenic acid	myo-Inositol 2-phosphate	Nicotinamide hypoxanthine dinucle	Propiconazole	Thiabendazole
2'-Deoxycytidine	3-Hydroxykynurenine	Acetoacetyl CoA	cIMP	γ-Glu-Arg	Ibuprofen	myo-Inositol 3-phosphate6	Nicotinamide N-oxide	Propionic acid	Thiamine
2'-Deoxyguanosine	3-Hydroxyoctanoic acid	Acetohydrazide	Cinnabarinic acid	γ-Glu-Asn	IDP	Myosmine	Nicotinamide riboside	Propionyl CoA	Thiamine diphosphate
2'-Deoxyinosine	3-Hydroxyphenylacetic acid	Acetohydroxamic acid	Cinnamoylglycine	γ-Glu-Asp	Ile	Myristic acid	Nicotine	Propionylcarnitine	Thiamine phosphate
2'-Deoxyuridine	3-Hydroxypicolinic acid	Acetyl CoA	Ciprofloxacin	γ-Glu-Citrulline	Ile-Gly	Myristoleic acid	Nicotinic acid	Propionylglycine	Thiamine triphosphate
2'-O-Methylcytidine	3-Hydroxyproline	Acetylcholine	cis,cis-Muconic acid	γ-Glu-Cys	Ile-Pro-Pro	N ,N-Dimethylaniline5	Nicotinic acid mononucleotide	Propranolol	Thiaproline
2,3-Diaminopropionic acid	3-Hydroxypropionic acid	Acetylenedicarboxylic acid	cis-4-Hydroxyproline	γ-Glu-Gln	Ile-Tyr	N ,N-Dimethylglycine	NMN	Propylthiouracil	Thiazole
2,3-Dihydroxybenzoic acid	3-Hydroxysebacic acid	Acetylphosphate	cis-Aconitic acid	γ-Glu-Glu	Imidazole-4-acetic acid	N ,N-Dimethylhistidine	Noradrenaline	Prostaglandin E2	Thiodiglycolic acid
2,3-Diphosphoglyceric acid	3-Indoleacetonitrile	Acetylpyrazine	Citraconic acid	γ-Glu-Gly	Imidazole-4-methanol	N 1,N 11-Diethylnorspermine N 1,N	Normetanephrine	Prostaglandin F2α	Thiourea
2,4,4'-Trichloro-2'-hydroxydiphenyl (	3-Indolebutyric acid	Adenine	Citramalic acid	γ-Glu-His	Imidazolelactic acid	N 1,N 12-Diacetylspermine P 1, P 4-	Nornicotine	Protocatechuic acid	Thr
2,4,5-Trichlorophenoxyacetic acid	3-Indoxylsulfuric acid	Adenosine	Citric acid	γ-Glu-Ile	Iminodiacetic acid	N 1,N 8-Diacetylspermidine	Norspermidine	PRPP	Thr-Asp
2,4-Diaminobutyric acid	3-Iodothyronamine	Adenosine 5'-[γ-thio]triphosphate	Citrulline	γ-Glu-Leu	IMP	N 1-Acetylspermidine N 8-Acetylspe	Norvaline	Pseudoephedrine	Thr-Hyp
2,4-Dichlorobenzoic acid	3-Iodotyrosine	Adenosine 5'-phosphosulfate	Clonidine	γ-Glu-Lys	Indole-3-acetaldehyde	N 1-Acetylspermine	O ,O-Diethyl thiophosphate	Pseudopelletierine	threo-3-Hydroxyaspartic acid
2,4-Dichlorophenoxyacetic acid	3-Isopropylmalic acid	Adenylosuccinic acid	CMP	γ-Glu-Met	Indole-3-acetaldoxime	N 1-Glutathionylspermidine disulfid	O-Acetylcarnitine	Pseudouridine	threo-β-Methylaspartic acid
2,4-Dihydroxybenzoic acid	3-Mercapto-2-oxopropanoate	Adipic acid	CMP-N-acetylneuraminate	γ-Glu-Ornithine	Indole-3-acetamide	N 2,N 2-Dimethylguanosine	O-Acetylhomoserine	Psychosine	Threonic acid
2,4-Dihydroxypyrimidine-5-carboxyl	3-Mercaptolactic acid	ADMA	CoA	γ-Glu-Phe	Indole-3-acetic acid	N 2-Acetylaminoadipic acid	O-Acetylserine	Pterin	Thymidine
2,4-Dimethylaniline5 2,6-Dimethyla	3-Methoxy-4-hydroxyphenylethylen	ADP	Corosolic acid	γ-Glu-Ser	Indole-3-carboxylic acid	N 2-Methylguanine N 1-Methylguan	o-Aminophenol	Purine	Thymine
2,5-Dihydroxybenzoic acid	3-Methoxyanthranilic acid	ADP-glucose1	Creatine	γ-Glu-Taurine	Indole-3-ethanol	N2-Methyllysine Nε-6-Methyllysine	o-Chlorobenzoic acid	Purine riboside	Tiglic acid
2,5-Dimethylpyrazine	3-Methoxytyramine	ADP-ribose	Creatinine	γ-Glu-Thr	Indole-3-glyoxylic acid	N 2-Phenylacetylglutamine	o-Coumaric acid	Putrescine	Tiglylcarnitine
2,5-Pyrroledione	3-Methoxytyrosine	Adrenaline	Crotonic acid	γ-Glu-Trp	Indole-3-lactic acid	N 2-Succinylornithine	o-Fluorobenzoic acid	Pyrazinamide	Timolol
2,6-Diaminopimelic acid	3-Methyl-2-oxovaleric acid7 4-Meth	Agmatine	Crotonic acid betaine	γ-Glu-Tyr	Indole-3-propionic acid	N 4-Acetylcytidine	o-Hydroxybenzoic acid	Pyrazinoic acid	Tioconazole
2,6-Diethylaniline	3-Methyladenine	Ala	Crotonoyl CoA	γ-Glu-Val	Indole-3-pyruvic acid	N 5-Ethylglutamine	o-Hydroxyhippuric acid	Pyrazole	Tobramycin
2,6-Dihydroxybenzoic acid	3-Methyladipic acid	Ala-Ala	cTMP	γ-Glu-Val-Gly	Indoleacrylic acid	N 6,N 6,N 6-Trimethyllysine	o-Hydroxyphenylacetic acid	Pyridine	Tolazoline
2-(3-Hydroxyphenyl)propionic acid	3-Methylcrotonyl CoA	Ala-Hyp	CTP	γ-Glutamyl-S-Allylcysteine	Inosine	N 6,N 6-Dimethyllysine	o-Methoxyphenyl sulfate	Pyridine-2-carboxylic acid butyl este	Tolmetin
2-(4-Hydroxyphenyl)propionic acid	3-Methylcrotonylglycine	Albendazole	Cumic acid	GDP	Isatin	N 6-(□E2-Isopentenyl)adenine	o-Phenanthroline	Pyridoxal	Topiramic acid
2-(Hydroxymethyl)benzoic acid	3-Methylcytidine	Albizziine	Cyanuric acid	GDP-fucose1	Isethionic acid	N 6-Acetyllysine	O-Phosphoserine	Pyridoxal 5-phosphate	Tranexamic acid
2-Amino-2-(hydroxymethyl)-1,3-pro	3-Methylguanine	Allantoic acid	Cyclohexanecarboxylic acid	GDP-glucose	Ismelin	N 6-Benzyladenine	O-Succinylhomoserine	Pyridoxamine	trans-2-Phenylcyclopropylamine
2-Amino-2-methyl-1,3-propanediol	3-Methylhistamine	Allantoin	Cyclohexylamine	GDP-mannose	Isoamylamine	N 6-Formyllysine	O-Sulfoserine	Pyridoxamine 5'-phosphate	trans-Cinnamic acid
2-Amino-2-methyl-1-propanol	3-Methylhistidine	Alliin	Cycloserine	Geranyl diphosphate	Isobutylamine	N 6-Methyl-2'-deoxyadenosine	o-Toluic acid	Pyridoxine	trans-Crocetin
2-Amino-2-methylbutyric acid	3-Methylxanthine	allo-Threonine	Cyprodinil	Gibberellic acid	Isobutyric acid	N 6-Methyladenine	Octanoic acid	Pyridoxol 5'-phosphate	trans-Glutaconic acid
2-Amino-3-phosphonopropionic acid	3-Nitropropionic acid	Allocryptopine	Cys	Gln	Isobutyryl CoA	N 6-Methyladenosine	Octanoyl CoA	Pyrimidine	trans-Zeatin
2-Aminoadipic acid	3-Nitrotyrosine	Alloisoleucine	Cys-Gly	Glu	Isobutyrylcarnitine	N α-Benoylarginine ethylester	Octanoylcarnitine	Pyrocatechol sulfate	trans-Zeatin riboside
2-Aminobenzenesulfonic acid	3-Phenyllactic acid	Allysine	Cystathionine	Glu-Glu	Isobutyrylglycine	N α-Succinylarginine	Octopamine	Pyrogallol	Trehalose 6-phosphate
2-Aminobenzimidazole	3-Phenylpropionic acid	α-Chaconine	Cysteamine	Glucaric acid	Isocitric acid	N ρE(Carboxymethyl)arginine	Octopine	Pyroglutamine	TRH
2-Aminobutyric acid	3-Phosphoglyceric acid	α-Solanine	Cysteic acid	Gluconic acid	Isoferulic acid	N ρEMethylarginine	Octylamine	Pyrophosphate	Triadimefon
2-Aminoethylphosphonic acid	3-Sulfopropanediol	Amantadine	Cysteine glutathione disulfide	Gluconolactone	Isoglutamic acid	N-(1-Deoxy-1-fructosyl)glycine	Oleandomycin	Pyrraline	Tridecanoic acid
2-Aminoisobutyric acid	3-Ureidoisobutyric acid	Amikacin	Cysteinesulfinic acid	Glucosamine	Isoniazid	N-(1-Deoxy-1-fructosyl)leucine	Ophthalmic acid	Pyrrole	Trientine
2-Aminooctanoic acid	3-Ureidopropionic acid	Amiloride	Cystine	Glucosamine 6-phosphate	Isonicotinamide	N-(1-Deoxy-1-fructosyl)threonine	Ornithine	Pyrrole-2-carboxylic acid	Triethanolamine
2-C-Methylerythritol 4-phosphate	4,4'-Methylene bis(o-chloroaniline)	Aminoacetone	Cytidine	Glucosamine 6-sulfuric acid	Isonicotinic acid	N-(1-Deoxy-1-fructosyl)valine	Orotic acid	Pyrroloquinoline quinone	Trifluoperazine
2-Carboxybenzaldehyde	4,6-Dihydroxyquinoline	Aminomalonic acid	Cytosine	Glucosaminic acid	Isopropanolamine	N-(o-Toluoyl)glycine	Orotidine	Pyruvic acid	Trigalacturonic acid
2-Cyanopyridine	4-(β-Acetylaminoethyl)imidazole	Aminophylline	dADP	Glucose 1-phosphate	Isoproterenol	N-Acetyl-β-alanine	Orotidine 5'-monophosphate	Quinic acid	Trigonelline
2-Deoxyribonic acid	4-Acetamido-2-aminobutyric acid	Aminopyrine	DAMGO	Glucose 6-phosphate	Isoquinoline	N-Acetylalanine	Oxalic acid	Quinine	Trimethoprim
2-Deoxyribose 1-phosphate	4-Acetamidobutanoic acid	AMP	dAMP	Glucurone	Isovaleric acid	N-Acetylasparagine	Oxamic acid	Quinoline	Trimethylamine
2-Ethylhexanoic acid	4-Amino-3-hydroxybutyric acid	Amrinone	dATP	Glucuronic acid	Isovalerylalanine	N-Acetylaspartic acid	Oxypurinol	Quinolinic acid	Trimethylamine N-oxide
2-Ethylhydracrylic acid	4-Aminohippuric acid	Anabasine	Daunorubicin	Glutamic acid γ-methyl ester	Isovalerylcarnitine	N-Acetylaspartylglutamic acid	p-Aminobenzoic acid	Quisqualic acid	Trimethylsulfonium
2-Furoic acid	4-Aminoindole	Aniline	dCDP	Glutaric acid	Isovalerylglycine	N-Acetylcarnosine	p-Aminophenol	Retinoic acid	Tropic acid
2-Guanidinobenzimidazole	4-Aminophenylsulfone	Anserine	dCMP	Glutaryl CoA	Isoxanthopterin	N-Acetylcysteine	p-Anisic acid	Rhein	Tropine
2-Hydroxy-3-methylvaleric acid	4-Aminosalicylic acid	Anthranilic acid	dCTP	Glutarylcarnitine	Itaconic acid	N-Acetylgalactosamine	p-Chlorobenzoic acid	Ribavirin	Tropinone
2-Hydroxy-4-methylvaleric acid	4-Bromophenylalanine	Apigenin	Deamido-NAD+	Glutathione (GSH)	ITP	N-Acetylgalactosamine 4-sulfate	p-Chlorophenoxyacetic acid	Riboflavin	Trp
2-Hydroxyadipic acid	4-Guanidinobutyric acid	Arabinonic acid	Decanoic acid	Glutathione (GSSG)	Kanamycin A	N-Acetylglucosamine	p-Chlorophenylacetic acid	Ribose 1-phosphate	Tryptamine
2-Hydroxybutyric acid	4-Hydroxy-3-methoxyphenylglycol s	Arabinosylhypoxanthine	Decarboxylated S-Adenosylmethion	Gly	Kinetin	N-Acetylglucosamine 1-phosphate	p-Coumaric acid	Ribose 5-phosphate	Tryptophanamide
2-Hydroxyglutaric acid	4-Hydroxycyclohexylcarboxylic acid	Arg	Deethylatrazine	Gly-Asp	Kojic acid	N-Acetylglucosamine 6-phosphate	p-Coumaric acid sulfate	Ribostamycin	Tyr
2-Hydroxyisobutyric acid	4-Hydroxyhippuric acid	Arg-Glu	Deisopropylatrazine	Gly-Gly	Kynurenic acid	N-Acetylglucosylamine	p-Cresol glucuronide	Ribulose 1,5-diphosphate	Tyr-Arg
2-Hydroxyisovaleric acid	4-Hydroxyphenethyl alcohol	Arginine ethyl ester	Delphinidin 3,5-diglucoside	Gly-Gly-Gly	Kynurenine	N-Acetylglutamic acid	p-Cresol sulfate	Ribulose 5-phosphate	Tyr-Glu
2-Hydroxyoctanoic acid	4-Hydroxyphenylacetaldehyde	Argininic acid	Deoxyxylulose 5-phosphate	Gly-Hyp	Lactic acid	N-Acetylglutamine	p-Fluoroaniline	S-(2-Carboxyethyl)cysteine	Tyramine
2-Hydroxypyridine	4-Hydroxyphenylglycine	Argininosuccinic acid	Dethiobiotin	Gly-Leu	Lauric acid	N-Acetylglycine	p-Fluorobenzoic acid	S-Adenosylhomocysteine	Tyrosine methyl ester
2-Hydroxyvaleric acid	4-Hydroxyphenylglyoxylic acid	Ascorbate 2-glucoside	dGDP	Gly-Pro-Hyp	Lauroyl CoA	N-Acetylhistidine	p-Hydroxybenzoic acid	S-Adenosylmethionine	UDP
2-Isopropylmalic acid	4-Hydroxyquinoline	Ascorbate 2-phosphate	dGMP	Gly-Thr-Trp-Tyr	Lauroylcarnitine	N-Acetylisoleucine	p-Hydroxymandelic acid	S-Allylcysteine	UDP-galactose9 UDP-glucose9
2-Methylbutyrylglycine	4-Methyl-1,2,5-oxadiazol-3-amine	Ascorbate 2-sulfate	dGTP	Glyceraldehyde 3-phosphate	Lenticin	N-Acetylleucine	p-Hydroxyphenylacetic acid	S-Carboxymethylcysteine	UDP-glucuronic acid
2-Methylcholine	4-Methyl-5-thiazoleethanol	Ascorbic acid	Dichlorprop	Glyceric acid	Leu	N-Acetyllysine	p-Hydroxyphenylacetylglycine	S-Hexylglutathione	UDP-N-acetylgalactosamine
2-Methylglutaric acid	4-Methylaminobutyric acid	Asn	Diclofenac	Glycerol	Leu-Leu-Tyr	N-Acetylmannosamine	p-Hydroxyphenylpyruvic acid	S-Lactoylglutathione	UDP-N-acetylglucosamine
2-Methylserine	4-Methylpentanoic acid	Asp	Diethanolamine	Glycerol 2-phosphate	Leupeptin	N-Acetylmethionine	p-Methoxyphenylacetic acid	S-Methylcysteine	UMP
2-Methylthiazolidine-4-carboxylic ac	4-Methylpyrazole	Aspartame	Diethylaminomalonic acid	Glycerol 3-phosphate	Lidocaine	N-Acetylmuramic acid	p-Nitrophenyl phosphate	S-Methylcysteine-S-oxide	Undecanedioic acid
2-Oxoadipic acid	4-Methylthio-2-oxobutyric acid	ATP	Digalacturonic acid	Glycerophosphocholine	Lipoamide	N-Acetylneuraminic acid	p-Phenolsulfonic acid	S-Methylglutathione	Undecanoic acid
2-Oxobutyric acid	4-Oxohexanoic acid	Atrolactic acid	Dihydroorotic acid	Glycerophosphorylethanolamine	Loperamide	N-Acetylornithine	p-Sulfobenzoic acid	S-Methylmethionine	Uracil
2-Oxoglutaric acid	4-Oxopyrrolidine-2-carboxylic acid	Atropine	Dihydrouracil	Glycine anhydride	Lumazine	N-Acetylphenylalanine	p-Toluic acid	S-Nitrosoglutathione	Urea
2-Oxohexanoic acid	4-Oxovaleric acid	Azelaic acid	Dihydroxyacetone phosphate	Glycocholic acid	Lupulone	N-Acetylproline	Palmatine	S-Sulfocysteine	Ureidoglycolic acid
2-Oxoisovaleric acid	4-Pyridoxic acid	Azetidine 2-carboxylic acid	Diisopropanolamine	Glycolic acid	Lys	N-Acetylputrescine	Palmitoylcarnitine	Saccharopine	Uric acid
2-Oxooctanoic acid	4-Trimethylammoniobutanal	Barbituric acid	Dimethylallylpyrophosphate	Glycylsarcosine	Lysinamide	N-Acetylserine	Pantoic acid	Salsolinol	Uridine
2-Oxovaleric acid	5'-Deoxy-5'-methylthioadenosine	Benzamide	Dimethylaminoethanol	Glycyrrhetinic acid	m-Aminophenol	N-Acetyltaurine	Pantothenic acid	Sarcosine	Urocanic acid
2-Phenylethylamine	5,6,7,8-Tetrahydrobiopterin	Benzamidine	dIMP	Glyoxylic acid	m-Ethoxybenzoic acid	N-Acetylthreonine	Pantothenol	Scopolamine	Ursolic acid
2-Phosphoglyceric acid	5,6-Dihydroxyindole	Benzimidazole	Diphenylamine	GMP	m-Fluorobenzoic acid	N-Acetyltryptophan	Paraxanthine	SDMA	UTP
2-Quinolinecarboxylic acid	5,6-Dimethylbenzimidazole	Benzocaine	Diphenylcarbazide	Gramine	m-Hydroxybenzoic acid	N-Acetyltyrosine	Pargyline	Sebacic acid	Val
2-Thiopheneacetic acid	5-Amino-3,4-dihydro-2H-pyrrole-2-c	Benzoic acid	Disulfiram	GTP	m-Toluic acid	N-Acetylvaline	Paromomycin	Sedoheptulose 7-phosphate	Val-Pro-Pro
3',5'-ADP	5-Amino-4-oxovaleric acid	Benzoylformic acid	dITP	Guanidinosuccinic acid	Maleamic acid	N-α-Acetylarginine	Pelargonic acid	Selenocystine	Valeric acid
3',5'-Cyclic dAMP	5-Aminoimidazole-4-carboxamide ri	Benzyl viologen	DL-2-Methylbutyric acid	Guanidoacetic acid	Maleic acid	N-Amidinoglutamic acid	Penicillamine	Selenomethionine	Vanillic acid
3'-AMP	5-Aminoindole	Benzylamine	Dodecanedioic acid	Guanine	Malic acid	N-Butyrylglycine	Pentamidine	Semicarbazide	Vanillylmandelic acid
3'-Dephospho CoA	5-Aminovaleric acid	Benzylsuccinic acid	DOPA	Guanosine	Malonyl CoA	N-Carbamoylaspartic acid	Pentosidine	Sepiapterin	Verapamil
3'-UMP	5-Diphosphomevalonic acid	β-Ala	Dopamine	Guanosine 5'-[γ-thio]triphosphate	Malonylcarnitine	N-Carbamylglutamic acid	Perillic acid	Ser	Veratric acid
3,3',5-Triiodothyronine	5-Formylsalicylic acid	β-Ala-Lys	Dopamine 3-O-sulfate	Gulonolactone	Maltobionic acid	N-Ethylglycine	Phe	Ser-Glu	Xanthine
3,4-Dehydroproline	5-Hydroxy-2-indolecarboxylic acid	β-Asp-Leu	Dopamine 4-sulfate	H-Asp(Gly-OH)-OH	Mandelic acid	N-Ethylmaleimide	Phenaceturic acid	Serotonin	Xanthopterin
3,4-Dihydroxybenzylamine	5-Hydroxyindoleacetic acid	β-Carboline	dTDP	H-Asp(Lys-OH)-OH	Mannosamine	N-Formylanthranilic acid	Phenazopyridine	Shikimate 3-phosphate	Xanthosine
3,4-Dihydroxyhydrocinnamic acid	5-Hydroxyisophthalic acid	β-Cyanoalanine	dTDP-glucose	Harmaline	Melamine	N-Formylaspartic acid	Phenol	Shikimic acid	Xanthurenic acid
3,4-Dihydroxymandelic acid	5-Hydroxylysine	β-Hydroxyisovaleric acid	dTMP	Harman	Melatonin	N-Formylglycine	Phenol sulphate	Simazine	XMP
3,4-Dihydroxyphenylacetic acid	5-Hydroxymethyluracil	β-Hydroxypyruvic acid	dTTP	Heptanoic acid	Mesaconic acid	N-Formylkynurenine	Phenoxyacetic acid	Sinapic acid	XTP
3,4-Dihydroxyphenylglycol	5-Hydroxypentanoic acid	β-Leucine	dUDP	Hercynine	Mesalamine	N-Formylmethionine	Phenoxybenzamine	Sisomicin	Xylulose 5-phosphate
3,5-Dihydroxybenzoic acid	5-Hydroxytryptophan	β-Tyr	dUMP	Hexamine	meso-Tartaric acid	N-Formylphenylalanine	Phentolamine	Sorbitol 6-phosphate	
3,5-Diiodotyrosine	5-Methoxy-N,N-dimethyltryptamin	Betaine	dUTP	Hexanoic acid	Met	N-Glycolylneuraminic acid	Phenyl phosphate	Spectinomycin	
3,5-Dinitrosalicylic acid	5-Methoxyindoleacetic acid	Betaine aldehyde	Ectoine	Hexazinone	Met-Val-Pro	N-Hexanoylglycine	Phenylephrine	Spermidine	
3-(2,3-Dihydroxyphenyl) propionic a	5-Methoxytryptamine	Betonicine	Eflornithine	Hexylamine	Met-Val-Pro-Pro	N-Lactoylethanolamine	Phenylethanolamine	Spermine	
3-(2-Hydroxyphenyl)propionic acid	5-Methyl-2'-deoxycytidine	Biocytin	Ephedrine	Hippuric acid	Metamitron	N-Lactoylphenylalanine	Phenylhydrazine	Stachydrine	
3-(3,4-Dihydroxyphenyl)-2-methylal	5-Methylcytidine	Biopterin	Ergothioneine	His	Metanephrine	N-Methylalanine	Phenylpropionylglycine	Streptomycin sulfate	
3-(3-Hydroxyphenyl)propionic acid	5-Methylcytosine	Biotin	Erythrose 4-phosphate	His-Glu	Metanilic acid	N-Methylaniline	Phenylpyruvic acid	Subaphylline	

Note: The analytes listed constitute the targeted CE–MS panel provided by HMT. In the quantitative output, metabolites are reported only if a corresponding peak is detected in at least one sample; metabolites with no detected peak across all samples are not reported.
