# Supplementary figures and images for: Sixty Years After a Coal Mine Disaster: Serum Metabolomic Profiles in Older Adults with Long-Term Sequelae of Carbon Monoxide Poisoning: A Cross-Sectional Study
Source: Metabolites. 2026 Feb 12;16(2):126. doi: 10.3390/metabo16020126 (PMC12943369; doi:10.3390/metabo16020126)

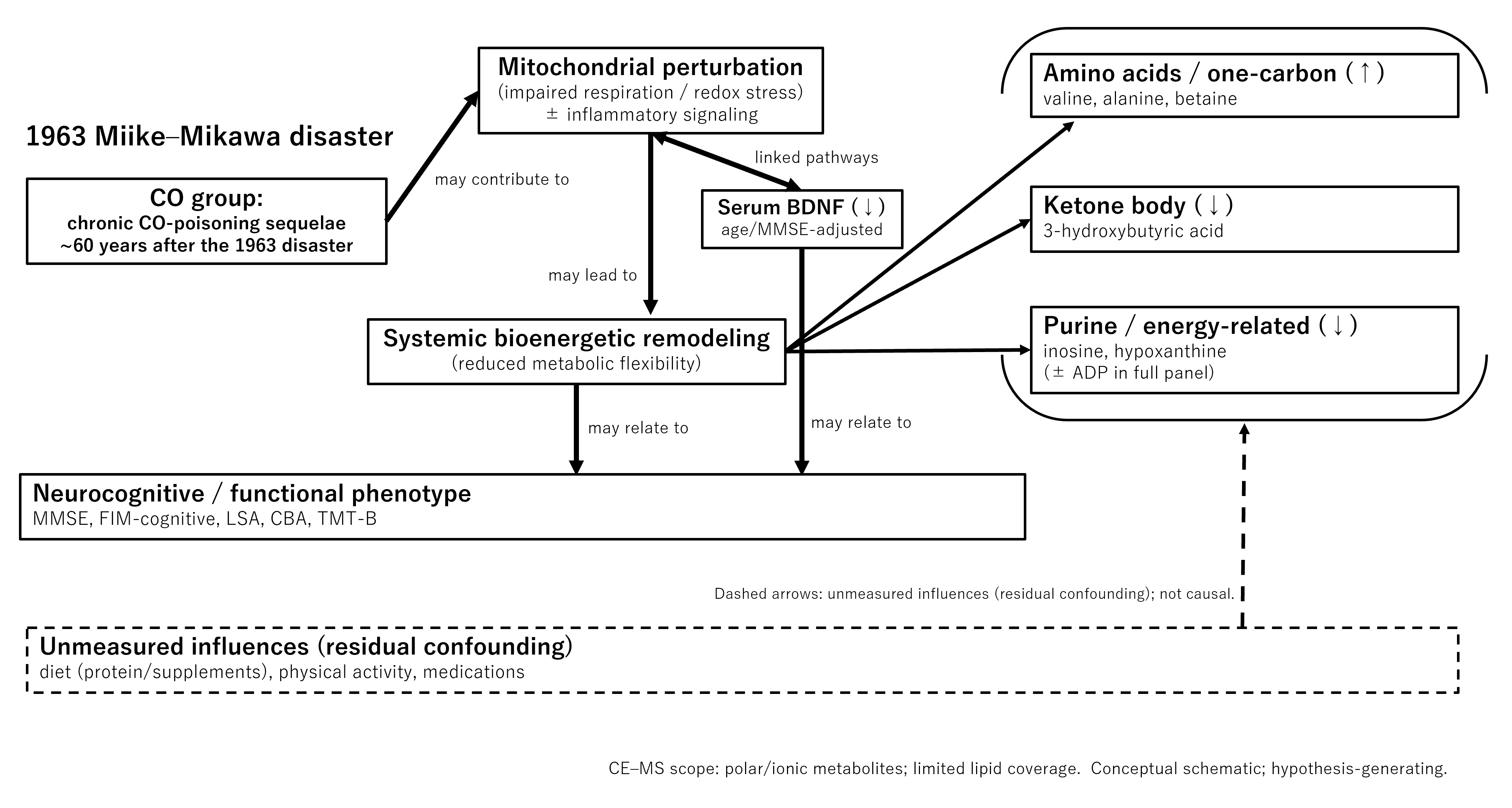

Supplement: Supplementary file 1 [file metabolites-16-00126-s001.zip › metabolites-4124646-supplementary/Revised Supplementary files/Figure S1_RGB.tif]
